# Blood–brain barrier disruption and sustained systemic inflammation in individuals with long COVID-associated cognitive impairment

**DOI:** 10.1038/s41593-024-01576-9

**Published:** 2024-02-22

**Authors:** Chris Greene, Ruairi Connolly, Declan Brennan, Aoife Laffan, Eoin O’Keeffe, Lilia Zaporojan, Jeffrey O’Callaghan, Bennett Thomson, Emma Connolly, Ruth Argue, James F. M. Meaney, Ignacio Martin-Loeches, Aideen Long, Cliona Ni Cheallaigh, Niall Conlon, Colin P. Doherty, Matthew Campbell

**Affiliations:** 1 Smurfit Institute of Genetics, Trinity College Dublin, Dublin, Ireland; 2 Department of Neurology, Health Care Centre, St James’s Hospital, Dublin, Ireland; 3 The Irish Longitudinal Study on Ageing, School of Medicine, Trinity College Dublin, Dublin, Ireland; 4 Clinical Research Facility, St James’s Hospital, Dublin, Ireland; 5 Thomas Mitchell Centre for Advanced Medical Imaging (CAMI), St. James’s Hospital & Trinity College Dublin, Dublin, Ireland; 6 Department of Intensive Care Medicine, Multidisciplinary Intensive Care Research Organization, Trinity Centre for Health Sciences, St James’s University Hospital, Dublin, Ireland; 7 Trinity Translational Medicine Institute, Trinity College Dublin, St James’s Hospital, Dublin, Ireland; 8 Department of Immunology, St James’s Hospital, Dublin, Ireland; 9 St James’s Hospital, Tallaght University Hospital, Trinity College Dublin Allied Researchers (STTAR) Bioresource, Trinity College Dublin, Dublin, Ireland; 10 Academic Unit of Neurology, Biomedical Sciences Institute, Trinity College Dublin, Dublin, Ireland; 11 FutureNeuro, Science Foundation Ireland Research Centre for Chronic and Rare Neurological Diseases, Royal College of Surgeons in Ireland, University of Medicine and Health Sciences, Dublin, Ireland

**Keywords:** Neuro-vascular interactions, Neuroimmunology, Diseases of the nervous system

## Abstract

Vascular disruption has been implicated in coronavirus disease 2019 (COVID-19) pathogenesis and may predispose to the neurological sequelae associated with long COVID, yet it is unclear how blood–brain barrier (BBB) function is affected in these conditions. Here we show that BBB disruption is evident during acute infection and in patients with long COVID with cognitive impairment, commonly referred to as brain fog. Using dynamic contrast-enhanced magnetic resonance imaging, we show BBB disruption in patients with long COVID-associated brain fog. Transcriptomic analysis of peripheral blood mononuclear cells revealed dysregulation of the coagulation system and a dampened adaptive immune response in individuals with brain fog. Accordingly, peripheral blood mononuclear cells showed increased adhesion to human brain endothelial cells in vitro, while exposure of brain endothelial cells to serum from patients with long COVID induced expression of inflammatory markers. Together, our data suggest that sustained systemic inflammation and persistent localized BBB dysfunction is a key feature of long COVID-associated brain fog.

## Abstract (web summary)

Long COVID is a major public health issue since 2020 and exhibits frequent neurological symptoms. Greene et al. propose that brain fog results from leaky brain blood vessels and a hyperactive immune system, shedding light on this phenomenon.

## Main

Coronavirus disease 2019 (COVID-19) is a clinical syndrome caused by severe acute respiratory syndrome coronavirus 2 (SARS-CoV-2). COVID-19 primarily affects the respiratory tract and can progress to respiratory compromise, severe acute respiratory distress syndrome (ARDS) and death^1,2^. ARDS due to COVID-19 has been associated with encephalopathy, agitation, confusion and corticospinal tract dysfunction. Such symptoms, however, including anosmia (although not to the extent seen in the first wave of COVID-19), may be expected in anyone recovering from a severe viral illness because of cytokine release, critical illness encephalopathy or medication^3^. Neurological sequelae of COVID-19, colloquially known as ‘brain fog’, are increasingly being reported and include headache, fatigue, malaise and altered levels of consciousness. For example, clinical observations of neurological complications in 236,379 patients in the 6 months after a COVID-19 diagnosis found that 33.62% of patients had demonstrated clinically important neurological or psychiatric dysfunction^4^. Neurological problems have been reported in other respiratory viral infections including influenza, coronavirus and metapneumovirus, with febrile or afebrile seizures, status epilepticus, encephalopathies and encephalitis being the most frequently reported^5^. However, there is still little understanding of the pathogenesis and long-term outcome of neurological problems after SARS-CoV-2 infection. SARS-CoV-2 gains cellular entry via its receptors angiotensin-converting enzyme 2 and transmembrane protease serine 2, but it may enter via other receptors, including neuropilin and vimentin, all of which are enriched in cells of the neurovascular unit^6–10^. There are, however, conflicting reports regarding the neuroinvasiveness of SARS-CoV-2 and indeed the cellular expression of the receptors^11–15^, suggesting that other mechanisms are responsible for the neurological problems reported. A recent study suggested persistence of viral RNA in multiple anatomic sites, including the brain, for up to 230 days after symptom onset, although these data were from postmortem donor tissues, which represent the sickest of individuals^16^.

Several lines of research suggested that breakdown to the integrity of the blood–brain barrier (BBB) and subsequent brain penetration of serum components and cytokines is responsible for the neurological manifestations after SARS-CoV-2 infection^17,18^. The BBB is formed by endothelial cells lining cerebral blood vessels and supported by surrounding cells including astrocytes, pericytes, microglia, neurons and the acellular basement membrane^19^. The barrier is characterized by an enrichment of interendothelial tight junction proteins, several luminal and abluminal transporters, and luminal efflux transporters, which together maintain separation of the blood and brain and tightly regulate molecular trafficking between the blood and brain and vice versa^20^.

There is clear evidence of microvascular injury in the brains of deceased patients with COVID-19, including fibrinogen leakage and thinning of the endothelial cell basal laminae in the olfactory bulb^14,21^. A more comprehensive evaluation of the same cohort using spatial transcriptomics revealed more detailed vascular and immunological features of microvessels in the brain, including serum protein extravasation, platelet accumulation and coagulation system activation^22^. Numerous studies also examined BBB-related changes and responses to SARS-CoV-2 infection or spike (S) protein treatment in postmortem tissue and animal models^14,15,21,23–28^. However, the cerebrovascular pathology in patients and the underlying mechanisms of pathology are still unclear, especially in individuals with long COVID.

The lack of a specific neurological signature of the disease is interesting because other zoonotic betacoronaviruses often produce robust and predictable neurological injury^29^. In humans, data from SARS and Middle East respiratory syndrome also showed that neurological injury in humans is rare, strongly suggesting that, normally, the BBB provides robust neuroprotection from viral CNS invasion in most patients^30^. The clinical manifestation of SARS-CoV2-induced BBB alterations in patients has not yet been reported.

In this study, we hypothesized that the neurological response to COVID may be due to BBB breakdown and subsequent extravasation of serum components. We show that BBB disruption is evident in patients with acute COVID with brain fog and a cohort of patients with persistent long COVID-associated brain fog. We suggest that measurement of BBB integrity may be a clinically useful biomarker of the neurological sequelae associated with COVID-19 in some patients. Added to this, targeted regulation of BBB integrity may also represent a new method of clinically managing patients with long COVID.

## Results

### Acute COVID-induced brain fog is associated with BBB dysfunction

**Fig. 1:**
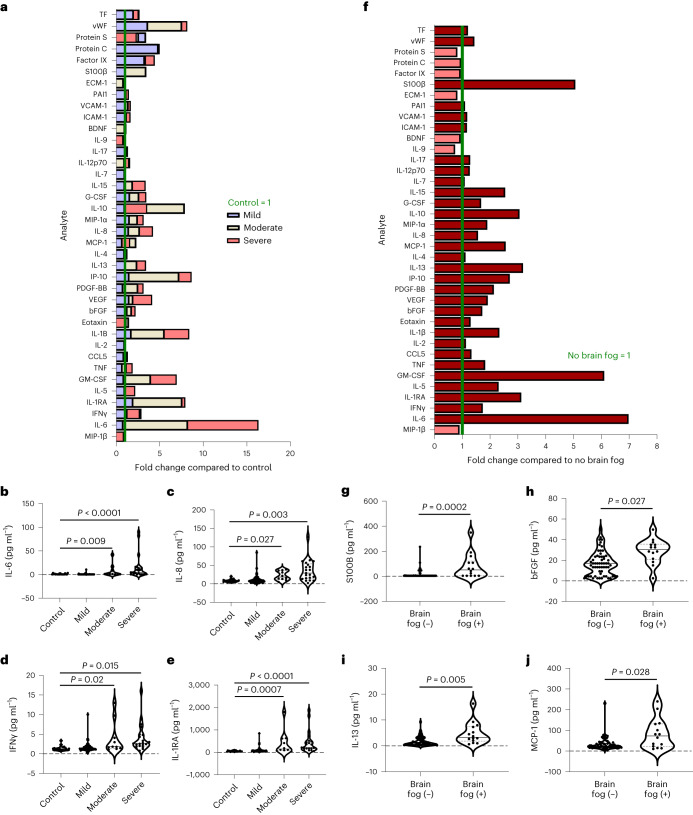
Inflammation and BBB permeability in cases infected with acute COVID-19. **a**, Analyte abundance plots showing serum concentrations of blood biomarkers in unaffected patients and patients with mild, moderate and severe SARS-CoV-2. Each cytokine was normalized to the respective mean cytokine level in unaffected individuals. BDNF, brain-derived neurotrophic factor; CCL5, C-C motif chemokine 5; MIP-1α, macrophage inflammatory protein-1 alpha; PAI1, plasminogen activator inhibitor-1; PDGF-BB, platelet-derived growth factor-BB; VEGF, vascular endothelial growth factor. **b**–**e**, Levels of IL-6 (*P* = 0.009 moderate versus control, *P* < 0.0001 severe versus control) (**b**), IL-8 (*P* = 0.027 moderate versus control, *P* = 0.003 severe versus control) (**c**), IFNγ (*P* = 0.02 moderate versus control, *P* = 0.015 severe versus control) (**d**) and IL-1RA (*P* = 0.0007 moderate versus control, *P* < 0.0001 severe versus control) (**e**) according to COVID severity. **f**, Analyte abundance plots showing serum concentrations of blood biomarkers in cases with brain fog versus cases without. Each cytokine was normalized to the respective mean cytokine level in individuals without brain fog. **g**–**j**, Levels of serum S100β (*P* = 0.0002) (**g**), bFGF (*P* = 0.027) (**h**), IL-13 (*P* = 0.005) (**i**) and MCP-1 (*P* = 0.028) (**j**) according to brain fog status. Data were analyzed using analysis of covariance (ANCOVA) adjusting for age, sex, COVID severity and comorbidities. The violin plots show the median (solid line) and interquartile (dashed lines) values; each data point represents one patient. Source data

**Extended Data Fig. 1:**
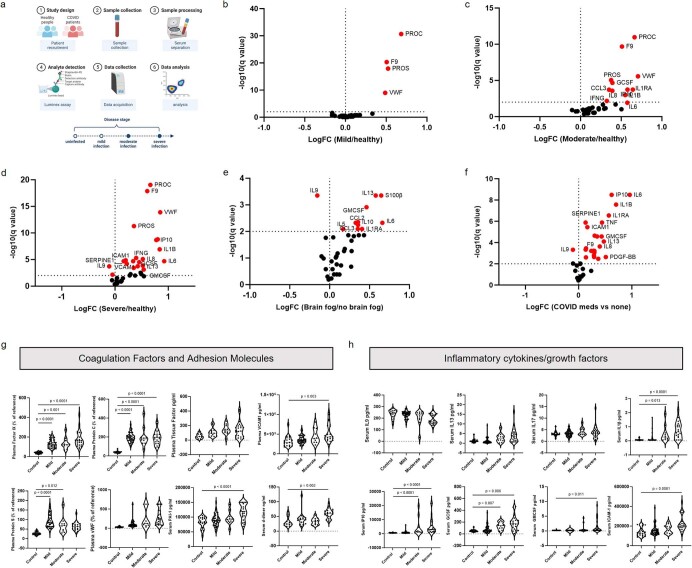
Disease severity is associated with changes in serum markers of inflammation. **a**) Study design for acute COVID cohort. **b**–**d**) Volcano plot of differentially expressed analytes in mild, moderate, and severe COVID patient’s vs healthy controls. **e**) Volcano plot of differentially expressed analytes between patients with vs without brain fog. **f**) Volcano plot of differentially expressed proteins between patients receiving vs not receiving COVID-directed medication. **g**) Levels of expression of Factor IX (p < 0.0001 Mild vs Control, p = 0.001 Moderate vs Control, p < 0.0001 Severe vs Control), Protein C (p < 0.0001 Mild vs Control, p < 0.0001 Moderate vs Control, p < 0.0001 Severe vs Control), Protein S (p < 0.0001 Mild vs Control, p = 0.012 Moderate vs Control), Tissue Factor, VCAM-1 (p = 0.0003 Severe vs Control), vWF, PAI-1 (p < 0.0001 Severe vs Control), D-dimer (p = 0.002 Severe vs Control). Data was analysed by ANCOVA adjusting for age and sex with Bonferroni correction for multiple comparisons. **h**) Levels of expression of IL-9, IL-13, IL-17, IL-1β (p = 0.013 Moderate vs Control, p < 0.0001 Severe vs Control), 10 kDa interferon gamma-induced protein (p < 0.0001 Moderate vs Control, p < 0.0001 Severe vs Control), G-CSF (p = 0.007 Moderate vs Control, p = 0.006 Severe vs Control), GM-CSF (p = 0.011 Severe vs Control), ICAM-1 (p = 0.0001 Severe vs Control). Data was analysed by ANCOVA adjusting for age and sex with Bonferroni correction for multiple comparisons. Violin plots show median (solid line) and interquartile values (dashed lines). Each datapoint represents one patient. All plots were generated in GraphPad PRISM. Multiple comparisons were controlled for by Benjamini-Hochberg correction. Schematic in a created with BioRender.com. Source data

**Extended Data Fig. 2:**
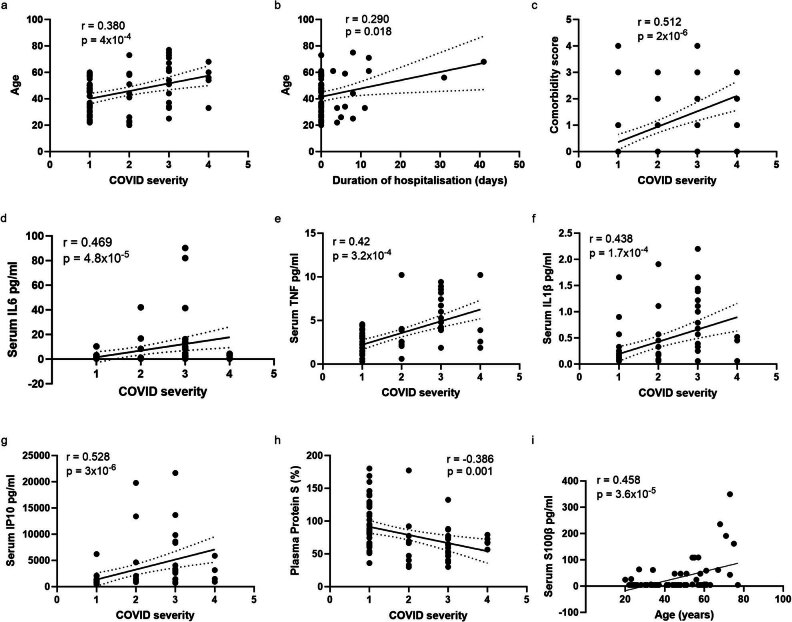
COVID severity is associated with blood levels of inflammatory cytokines. **a**) Pearson correlation between patient age and COVID severity. **b**) Pearson correlation between patient age and duration of hospitalisation. **c**) Pearson correlation between comorbidity score and COVID severity. **d**–**h**) Pearson correlation between levels of IL-6, TNF, IL-1B, IP-10 and Protein S with COVID severity. **i**) Pearson correlation between age and levels of S100β. Dashed lines represent 95 % confidence intervals. Data in d-i were analysed by partial correlations with age, sex, and comorbidities as covariates. Multiple comparisons were controlled for by Benjamini-Hochberg correction.

**Extended Data Fig. 3:**
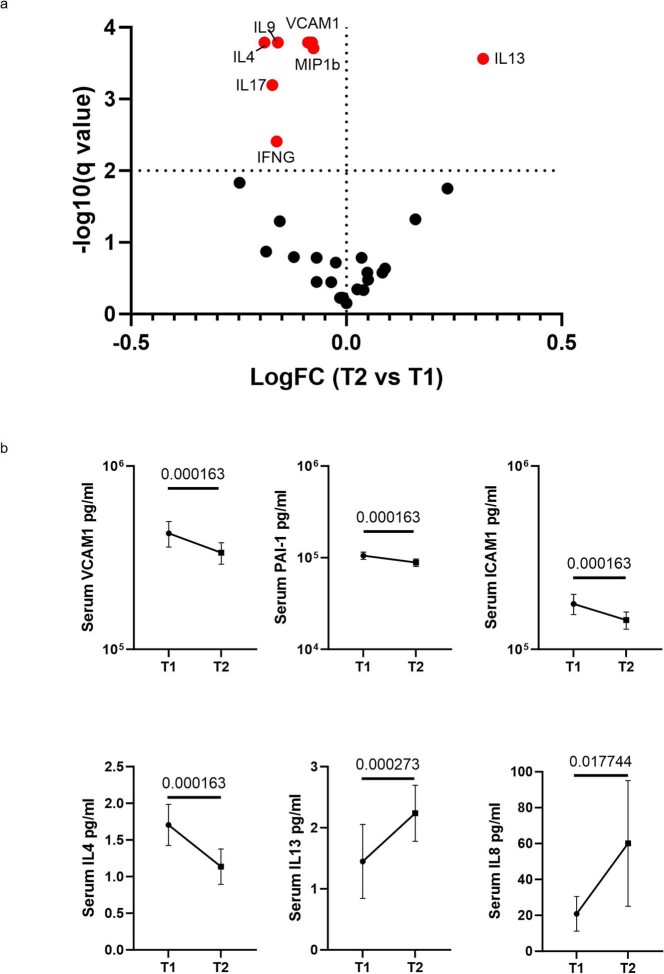
Longitudinal changes in serum analytes. **a**) Volcano plot of differentially expressed analytes between sample 1 (T1) and sample 2 (T2) following deterioration of clinical symptoms in hospitalised patients. **b**) Levels of sVCAM1, PAI-1, sICAM-1, IL-4, IL-13 and IL-8 between T1 and T2 (n = 27 paired samples). Error bars are mean ± s.d. For IL-8, ICAM-1, VCAM-1, PAI-1, VEGF, BDNF (n = 34 paired samples). Matched samples were compared with two-sided Wilcoxon signed-rank test. Multiple comparisons were controlled for by Benjamini-Hochberg correction. Source data

We collected serum and plasma samples from 76 inpatients with acute COVID-19 recruited as part of the St James’s Hospital, Tallaght University Hospital, Trinity College Dublin Allied Researchers Bioresource collection during the initial wave of COVID-19 in March and April 2020 (Extended Data Fig. 1a)^31^. Twenty-five unaffected control samples were collected before the COVID-19 pandemic. The mean age of the control and COVID samples was 44 and 44.7, respectively. The most frequent presenting symptoms included dyspnea (47), loss of smell and taste (46), cough (45), fatigue (40) and fever (36). Serum and plasma samples were screened with multiplex Luminex and ProcartaPlex panels for inflammatory, coagulation and BBB dysfunction markers. In total, we profiled 50 analytes in serum and plasma. The severity of COVID-19 was determined according to the World Health Organization (WHO) Severity Guidelines with 25 unaffected, 43 mild, 10 moderate and 23 severe. Of the 50 markers investigated, 4, 11 and 25 serum and plasma analytes were significantly different from controls in mild, moderate and severe groups, respectively after false discovery rate (FDR) correction and included several well-defined pro-inflammatory cytokines, including interferon-γ (IFNγ), interleukin-6 (IL-6), interleukin-1β (IL-1β), interleukin-1RA (IL-1RA), interleukin-8 (IL-8) and 10 kDa interferon gamma-induced protein (IP-10); growth factors, including granulocyte colony-stimulating factor (G-CSF) and granulocyte-macrophage colony-stimulating factor (GM-CSF); and markers of thrombosis and endothelial cell activation including plasminogen activator inhibitor-1 (PAI-1), protein C, protein S, Von Willebrand factor (vWF), factor IX, intercellular adhesion molecule 1 (ICAM-1) and vascular cell adhesion protein 1 (VCAM-1) (Fig. 1a–e and Extended Data Fig. 1b–d,h). Most markers were increased in moderate and severe cases except for coagulation markers, which were increased in all COVID groups (Fig. 1a and Extended Data Fig. 1g). Next, we determined if segregating according to brain fog status could reveal changes in the inflammatory profile of patients. Patients with brain fog had a higher mean age (53.7 versus 42.7) and were more likely to be hospitalized and require oxygen therapy; therefore, age, sex, comorbidities and severity of infection were included in the statistical model to identify differences between groups (Supplementary Table 1). Stratification of patients according to the presence or absence of brain fog revealed a general increase in most markers in the brain fog cohort (Fig. 1f), with significantly increased serum levels of protein S100β (Fig. 1g), a marker indirectly associated with BBB dysfunction. There were also increased levels of basic fibroblast growth factor (bFGF), interleukin-13 (IL-13) and monocyte chemoattractant protein-1 (MCP-1) in patients with brain fog (Fig. 1h–j). Correlation analysis revealed a significant correlation between WHO Severity of COVID-19 and age, duration of hospitalization and sum of comorbidities (Extended Data Fig. 2a–c). Therefore, partial correlations were performed with age, sex and all comorbidities as covariates, which revealed positive associations between serum concentrations of tumor necrosis factor (TNF), interleukin-6 (IL-6), IL-1β and IP-10 with COVID severity and an inverse association with plasma protein S (Extended Data Fig. 2d–h). We also found a significant association between serum S100β and age (Extended Data Fig. 2i). Of the 76 patients, 36 had a second blood sample drawn because of deterioration of clinical symptoms; thus, the serum concentrations of all analytes were assessed between time 1 and 2 (T1 and T2) to monitor disease progression. There was a significant decrease in serum concentrations of coagulation factors including PAI-1 and the cell adhesion molecules VCAM-1 and ICAM-1, while there was an increase in IL-13 and IL-8 between T1 and T2 (Extended Data Fig. 3a,b).

### BBB dysfunction is associated with long COVID-induced cognitive impairment

**Fig. 2:**
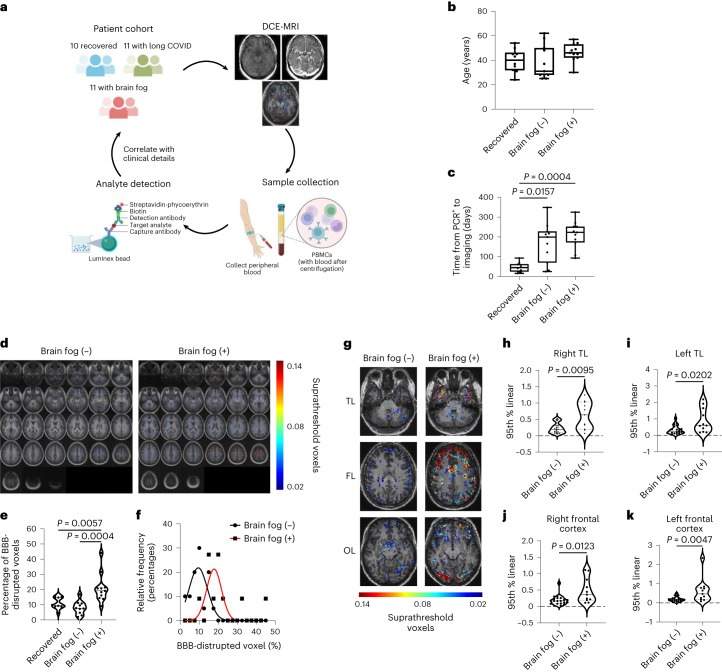
BBB disruption in long COVID-associated brain fog. **a**, Patient cohort for dynamic contrast-enhanced magnetic resonance imaging (DCE-MRI). **b**, Age distribution across cohorts (*n* = 10 recovered, *n* = 11 without brain fog (−), *n* = 11 with brain fog (+)). **c**, Time from COVID^+^ PCR test to scan across cohorts (*n* = 10 recovered, *n* = 11 without brain fog (−), *n* = 11 with brain fog (+)). Data were analyzed using a two-sided Kruskal–Wallis test with Dunn’s correction for multiple comparisons (*P* = 0.0157 without brain fog (−) versus recovered; *P* = 0.0004 with brain fog (+) versus recovered). **d**, Averaged BBB permeability maps in cases with and without brain fog. **e**, Quantification of the percentage of brain volume with leaky blood vessels in the cohort with brain fog compared to recovered cases (*P* = 0.0057) and cases without brain fog (*P* = 0.0004). Data were analyzed using a one-way analysis of variance with Tukey’s correction. **f**, Frequency distribution of the percentage of BBB-disrupted voxels in cases with and without brain fog. **g**, Representative BBB permeability maps at the level of the TLs, FLs and OLs showing enhanced BBB permeability in cases with brain fog. **h**–**k**, Quantification of regional BBB permeability in the right TL (*P* = 0.0095) (**h**), left TL (*P* = 0.0202) (**i**), right frontal cortex (*P* = 0.0123) (**j**) and left frontal cortex (*P* = 0.0047) (**k**). Data were analyzed using a two-sided Mann–Whitney *U*-test. The box plots display the minimum and maximum values (whiskers), median (solid line) and interquartile range (IQR) (upper and lower box). The violin plots show the median (solid line) and IQR (dashed lines); each data point represents one patient. Schematics in **a** were created with BioRender.com. Source data

Given the significantly increased serum concentrations of S100β, our data indicated that active and acute SARS-CoV-2 infection is associated with potential BBB dysfunction in individuals with neurological impairment. However, to directly visualize BBB function, we recruited ten recovered participants, 11 with long COVID and 11 with long COVID with brain fog who were diagnosed with COVID-19 during the first outbreak of disease in Ireland in April 2020 (Fig. 2a and Supplementary Table 2). All participants were recruited from St James Hospital Dublin and were PCR-confirmed cases of COVID-19. None of the patients in this cohort had received a vaccine and all had an initially mild course of disease that did not require hospitalization or antiviral treatment. We used a quick smell identification test (Q-SIT)-based method to determine objective anosmia status in participants and determined a strong correlation of reported anosmia status and Q-SIT score, providing an excellent readout of the utility of objective anosmia measurement in prolonged anosmia after COVID-19. Participants were grouped according to the presence or absence of self-reported cognitive issues termed ‘brain fog’ (brain fog (−) or brain fog (+)). Participants were considered as recovered when they reported no recurring symptoms following recovery from active SARS-CoV-2 infection. We hypothesized that COVID-19-associated cognitive impairment may be a strong predictor of BBB disruption in patients with COVID-19. There were no differences in age between each group (Fig. 2b). Imaging took place at a median time of 46, 175 and 211 days after PCR-confirmed SARS-CoV-2 infection for the recovered, long COVID and brain fog cohorts, respectively (Fig. 2c and Supplementary Table 2). Sixteen (50%) participants reported anosmia, which was confirmed using Q-SIT testing (average score 1 out of 3; 159 ± 88 days duration) at the time of scanning. Six participants with brain fog showed mild-to-moderate cognitive impairment on the Montreal Cognitive Assessment (MOCA) test (score 18–25) along with deficits in recall, executive functioning and word finding (Supplementary Table 2).

**Extended Data Fig. 4:**
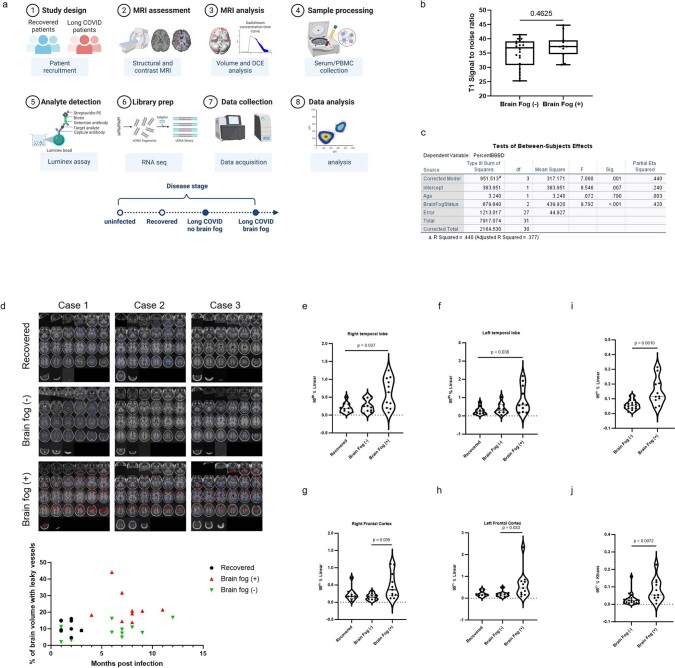
BBB disruption persists up to one year post SARS-CoV-2 infection in individuals with brain fog. **a**) Study design for Long COVID cohort. **b**) Signal to noise ratio in T1 weighted scans (n = 21 Brain Fog (-), n = 11 Brain Fog (+)). **c**) Summary statistics for the effects of age and brain fog on BBB permeability (effect of brain fog status on % BBB disruption p = 0.0006). **d**) Representative DCE-MRI scans from 3 recovered, Long COVID and brain fog participants. **e**–**h**) Quantification of regional BBB permeability in the right temporal lobe (p = 0.037 Brain Fog (+) vs Recovered), left temporal lobe (p = 0.036 Brain Fog (+) vs Recovered), right frontal cortex (p = 0.039 Brain Fog (+) vs Brain Fog (-)) and left frontal cortex (p = 0.033 Brain Fog (+) vs Brain Fog (-). Data analysed by two-sided Kruskal-Wallis test with Bonferroni correction. **i**) Whole brain 95th percentile values in participants with or without brain fog (p = 0.001). Data was analysed by two-sided Mann-Whitney test. **j**) 95th percentile Ktrans values in participants with or without brain fog (p = 0.0072). Data was analysed by two-sided Mann-Whitney test. Box plots display min and max values (whiskers), median values (solid line) and interquartile range (upper/lower box). Violin plots show median (solid line) and interquartile values (dashed lines). Schematic in a created with BioRender.com. Source data

**Extended Data Fig. 5:**
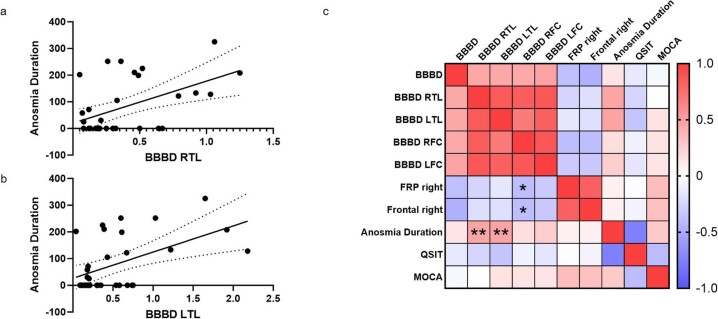
BBB disruption in the temporal lobes correlates with duration of anosmia. **a**, **b**) Spearman correlations between regional BBB disruption in the temporal lobes and the duration of anosmia. Dashed lines represent 95 % confidence intervals. **c**) Correlation heatmap between regional BBB disruption, anosmia status and cognitive performance as assessed by the Montreal Cognitive Assessment (MOCA). Data was analysed by Spearman partial correlations adjusted for age and sex. Source data

While standard diagnostic MRI scans showed no clinically relevant pathological findings in any participant, DCE-MRI imaging revealed significantly increased whole-brain leakage in patients with long COVID with brain fog (Fig. 2d–f), with an increased percentage of brain volume with leaky blood vessels in the cohort with brain fog compared to the cohort without brain fog. Stratifying the cohort into recovered, long COVID without brain fog and long COVID with brain fog revealed significantly increased BBB permeability in the cohort with brain fog compared to recovered patients and patients with long COVID without brain fog. Region of interest analysis identified significantly increased leakage in the right and left temporal lobes (TLs) and right and left frontal cortex (Fig. 2g–k). Stratifying the groups according to recovered, long COVID or brain fog revealed significantly increased BBB permeability in the group with brain fog only in the right and left TL and right and left frontal cortex (Extended Data Fig. 4). There was no difference in age between those with or without brain fog, with age not being associated with BBB dysfunction (Extended Data Fig. 4b,c). There was no association between BBB permeability and anosmia status, duration of anosmia, Q-SIT or MOCA scores (Extended Data Fig. 5a–c and Supplementary Table 3); however, regional BBB permeability in the right and left TLs was correlated with the duration of anosmia (Extended Data Fig. 5a,b).

### Long COVID-associated brain fog induces structural changes in the brain

**Fig. 3:**
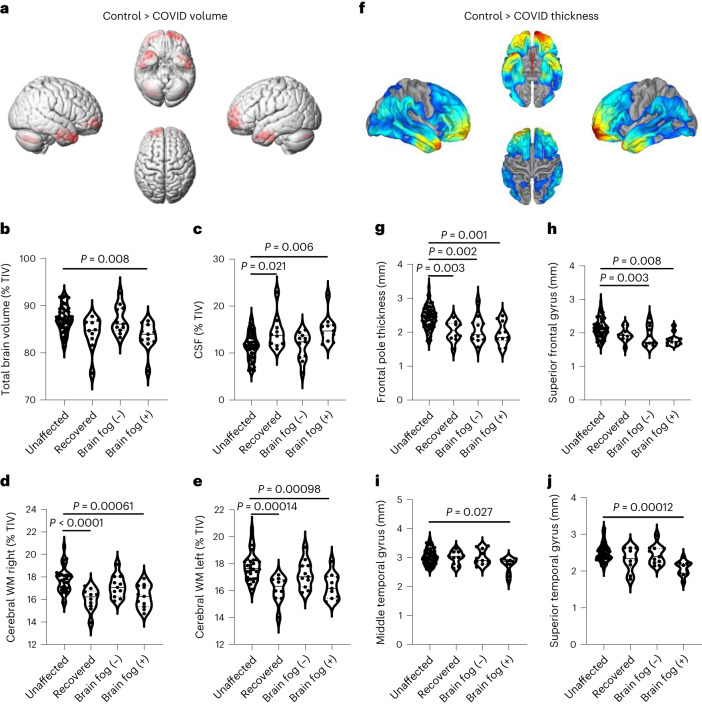
COVID-associated brain changes. **a**, Voxel-based morphometry map indicating brain regions with reduced volume in patients with previous SARS-CoV-2 infection. **b**–**e**, Group-wise comparison of total brain volume (*P* = 0.008 brain fog (+) versus control) (**b**), CSF volume (*P* = 0.021 recovered versus control; *P* = 0.006 brain fog (+) versus control) (**c**), right WM volume (*P* < 0.0001 recovered versus control; *P* = 0.00061 brain fog (+) versus control) (**d**) and left WM volume (*P* = 0.00014 recovered versus control; *P* = 0.00098 brain fog (+) versus control) (**e**) in unaffected individuals, recovered individuals and individuals with long COVID and brain fog. Data were analyzed using an ANCOVA, adjusting for age, sex and total intracranial volume (TIV), with Bonferroni correction. **f**, Surface-based morphometry map indicating brain regions with reduced cortical thickness in patients with previous SARS-CoV-2 infection. **g**–**j**, Group-wise comparison of frontal pole thickness (*P* = 0.003 recovered versus control; *P* = 0.002 brain fog (−) versus control; *P* = 0.001 brain fog (+) versus control) (**g**), superior frontal gyrus thickness (*P* = 0.003 brain fog (−) versus control; *P* = 0.008 brain fog (+) versus control) (**h**), middle temporal gyrus (*P* = 0.027 brain fog (+) versus control) (**i**) and superior temporal gyrus (*P* = 0.00012 brain fog (+) versus control) (**j**) in the unaffected, recovered, long COVID and brain fog cohorts. Data were analyzed using an ANCOVA adjusting for age and sex with Bonferroni correction. Maps were generated with Computational Anatomy Toolbox (CAT12) running in the Statistical Parametric Mapping (SPM12) software on MATLAB 2021a. The violin plots show the median (solid line) and IQR (dashed lines). Cohorts were compared with an unpaired *t*-test, with a family-wise error of less than 0.05, adjusted for age, sex and TIV. Volumetric and thickness region of interest measurements were obtained from volBrain. Source data

**Fig. 4:**
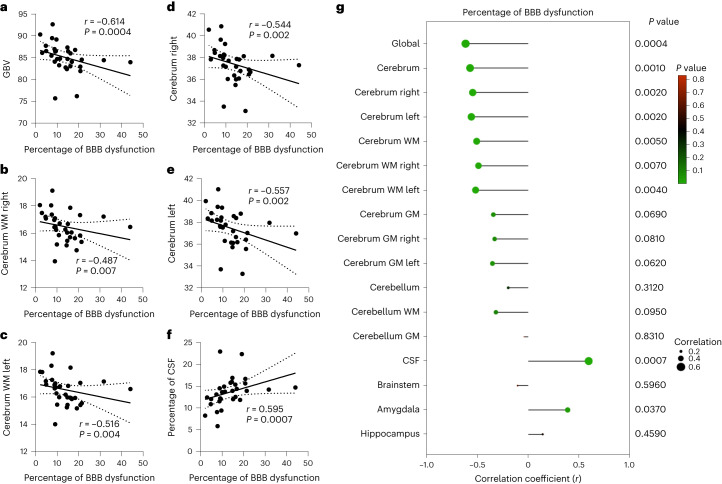
BBB permeability is associated with structural brain changes. **a**–**f**, Spearman partial correlation between the percentage of BBB-disrupted voxels and GBV (**a**), WM right volume (**b**), WM left volume (**c**), right cerebrum volume (**d**), left cerebrum volume (**e**) and CSF volume (**f**). The dotted lines represent the 95% confidence intervals (CIs). **g**, Plot of Spearman correlations between BBB permeability and brain volume measurements. Each data point represents one patient. Dot size corresponds to the Spearman correlation coefficient, while color represents the *P* value. Spearman partial correlation analysis for all panels was adjusted for age, sex and TIV. Source data

To explore if there were structural brain changes accompanying increased BBB permeability in our cohorts, we conducted volume and thickness measurements on recovered, long COVID and 60 age-matched healthy controls from the publicly available IXI dataset (Supplementary Table 2) and examined global brain volume (GBV), cerebrospinal fluid (CSF) volume and the right and left volumes of the cerebral and cerebellar white matter (WM) and gray matter (GM), and the brainstem, hippocampus and amygdala. Comparing all individuals with previous COVID infection to unaffected controls revealed volumetric deficits predominantly in the FLs and TLs and increases in the lateral ventricles and occipital lobes (OLs) (Fig. 3a), while group-wise comparisons of macrostructures revealed decreased GBV in patients with brain fog along with significantly reduced cerebral WM volume in both hemispheres in the recovered and brain fog cohorts along with reduced cerebellar WM volume in the recovered, long COVID and brain fog cohorts (Fig. 3b–d and Supplementary Table 3). There was a significantly increased CSF volume in the cohort with brain fog only (Fig. 3e and Supplementary Table 3). Cortical thinning was also evident predominantly in the TLs and frontal lobes (FLs) when analyzing all patients with previous SARS-CoV-2 infection compared to unaffected controls (Fig. 3f). When comparing groups, there was reduced thickness in the frontal pole in the recovered, long COVID and brain fog cohorts; the superior frontal gyrus in the cohorts with long COVID and brain fog; the middle temporal gyrus in the cohort with brain fog only; and the superior temporal gyrus in the cohort with brain fog only (Fig. 3g–j). Spearman partial correlations revealed significant negative associations between the number of BBB-disrupted voxels with GBV, right and left WM volume, and right and left cerebral volume and was positively associated with CSF volume (Fig. 4a–g). Regionally, we observed a negative correlation between BBB disruption in the right frontal cortex with the volume of the right frontal cortex and right frontal pole (Extended Data Fig. 5c).

### Immunovascular dysregulation in long COVID blood samples

**Fig. 5:**
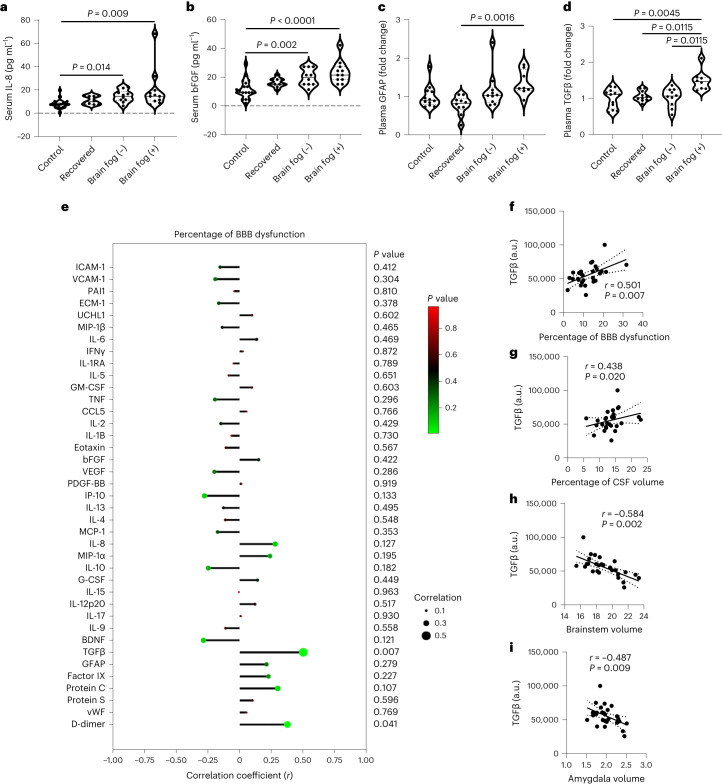
Plasma TGFβ is associated with increased BBB permeability. **a**–**d**, Serum and plasma concentrations of IL-8 (*P* = 0.014 brain fog (−) versus control; *P* = 0.009 brain fog (+) versus control) (**a**), bFGF (*P* = 0.002 brain fog (−) versus control; *P* < 0.0001 brain fog (+) versus control) (**b**), GFAP (*P* = 0.0016 brain fog (+) versus recovered) (**c**) and TGFβ (*P* = 0.0045 brain fog (+) versus control; *P* = 0.0115 brain fog (+) versus recovered; *P* = 0.0115 brain fog (+) versus brain fog (−)) (**d**) between each cohort. **a**,**b**, Data were analyzed using an ANCOVA adjusted for age and sex with Bonferroni correction. **c**,**d**, Data were analyzed using a two-tailed Kruskal–Wallis test with Dunn’s correction. **e**, Correlation plot between analyte levels and BBB permeability. **f**–**i**, Spearman correlation between levels of TGFβ and percentage of BBB dysfunction (**f**), percentage of CSF volume (**g**), brainstem volume (**h**) and amygdala volume (**i**). The dashed lines represent the 95% CIs. The violin plots show the median (solid line) and IQR (dashed lines). Each data point represents one patient. **a**–**d**, Kruskal–Wallis test. **f**–**i**, Spearman partial correlation analysis controlling for age, sex and TIV. Multiple comparisons were Benjamini–Hochberg-corrected, with *P* < 0.026 considered discoveries. Source data

**Extended Data Fig. 6:**
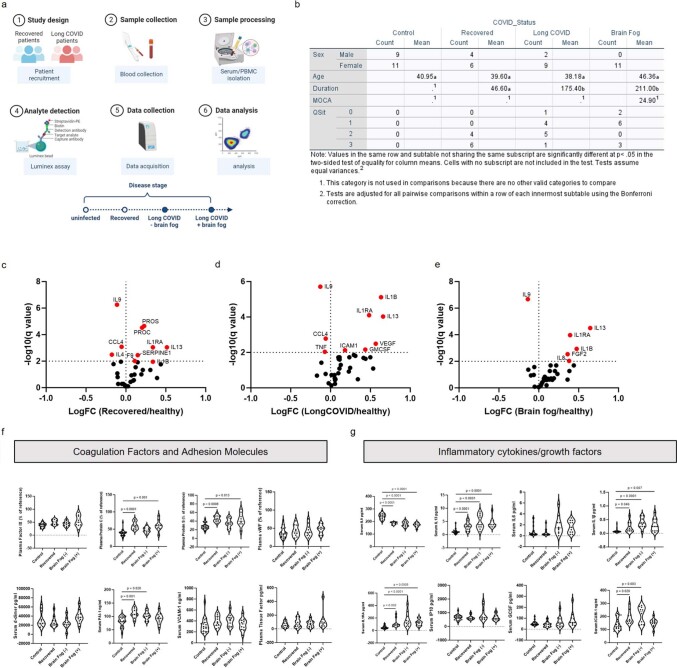
Systemic inflammatory changes in recovered and Long COVID patients. **a**) Study design for Long COVID cohort. **b**) Demographics of all cohorts. Data was analysed by One-Way ANOVA with Bonferroni correction for multiple comparisons. **c**–**e**) Volcano plots of differentially expressed analytes in recovered, Long COVID and brain fog cohorts compared to healthy controls. Multiple comparisons were controlled for by Benjamini-Hochberg correction. **f**) Level of expression of Factor IX, Protein C (p = 0.0001 Recovered vs Control, p = 0.001 Brain Fog (+) vs Control), Protein S (p = 0.0008 Recovered vs Control, p = 0.013 Brain Fog (+) vs Control), vWF, D-dimer, PAI-1 (p = 0.001 Recovered vs Control, p = 0.017 Brain Fog (-) vs Control), VCAM-1 and Tissue Factor according to disease status. Data was analysed by ANCOVA adjusting for age and sex with Bonferroni correction for multiple comparisons. **g**) Levels of expression of IL-9 (p < 0.0001 Recovered vs Control, p < 0.0001 Brain Fog (-) vs Control, p < 0.0001 Brain Fog (+) vs Control), IL-13 (p < 0.0001 Recovered vs Control, p < 0.0001 Brain Fog (-) vs Control, p < 0.0001 Brain Fog (+) vs Control), IL-6, IL-1β (p = 0.046 Recovered vs Control, p < 0.0001 Brain Fog (-) vs Control, p = 0.007 Brain Fog (+) vs Control), IL-1RA (p = 0.002 Recovered vs Control, p < 0.0001 Brain Fog (-) vs Control, p = 0.0005 Brain Fog (+) vs Control), IP-10, G-CSF and ICAM-1 (p = 0.020 Recovered vs Control, p = 0.003 Brain Fog (-) vs Control) according to disease status. Data was analysed by ANCOVA adjusting for age and sex with Bonferroni correction for multiple comparisons. Violin plots show median (solid line) and interquartile values (dashed lines). Each datapoint represents one patient. Schematic in a created with BioRender.com. Source data

Next, we analyzed blood-based biomarkers of neuroinflammation and BBB dysfunction in the recovered and long COVID cohort using multiplex Luminex panels as done for the acute cohort (Extended Data Fig. 6a). We examined 50 markers of BBB integrity and inflammation. Several markers were increased across all groups compared to controls including IL-1RA, IL-1β, bFGF and IL-13, while IL-9 was the only cytokine decreased in all groups (Fig. 5a–c and Extended Data Fig. 6). Glial fibrillary acidic protein (GFAP) was increased in the cohort with brain fog compared to recovered individuals while only transforming growth factor-β (TGFβ) was selectively increased in the cohort with brain fog compared to the cohort with long COVID without brain fog (Fig. 5c,d). There were also changes in plasma levels of coagulation markers with significantly increased levels of proteins C and S in the recovered cohort and the cohort with brain fog (Extended Data Fig. 6g). Next, we performed correlation analysis adjusting for age and sex to identify any associations between neuroinflammatory and BBB dysfunction markers with BBB permeability assessed using DCE-MRI. Levels of TGFβ and D-dimer were significantly associated with the percentage of voxels with abnormal leakage but only TGFβ was significant after correcting for multiple comparisons (Fig. 5e). The levels of TGFβ were significantly associated with GBV, CSF volume, brainstem volume and amygdala volume (Fig. 5f–i).

### White blood cells from patients with COVID-19 activate brain endothelial cells

**Extended Data Fig. 7:**
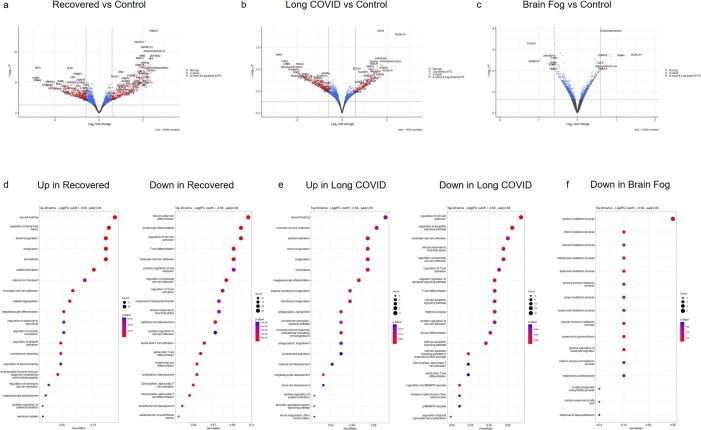
Transcriptional characterisation of PBMCs across all patient groups. **a**–**c**) Volcano plots of differentially expressed analytes in recovered, Long COVID and brain fog patient’s vs healthy controls. Data was analysed by Wald test with multiple comparisons controlled with Benjamini-Hochberg correction. **d**–**f**) Bubble plots of enriched Gene Ontology biological processes in each cohort. Bubble plots show top 20 enriched terms with absLog2FC cut-off < 0.59 and adjusted p-value < 0.05. Pathway enrichment p values were calculated in clusterProfiler with the enrichGO function with Benjamini-Hochberg adjustment and cutoff p-value of 0.05.

Given the prevalence of circulating markers indicative of BBB dysfunction and immune cell activation, we examined gene expression changes in peripheral blood mononuclear cells (PBMCs) isolated from unaffected (*n* = 7), recovered (*n* = 5) and patients with long COVID with (*n* = 6) or without (*n* = 5) brain fog using RNA sequencing (RNA-seq). Compared to unaffected individuals, there were 950 differentially expressed genes (DEGs) in recovered individuals, 481 in individuals with long COVID and 126 in individuals with brain fog in our cohorts (Extended Data Fig. 7a–c). Next, we performed gene ontology (GO) analysis. In the recovered cohort and the cohort without brain fog, upregulated terms included those related to the coagulation system, such as blood coagulation (for example, *F13A1*, *PROS1*), platelet activation (for example, *F2R*, *PF4*, *PF4V1*), platelet degranulation (for example, *SELP*, *VCL*, *CLU*) as well as megakaryocyte development, immunoglobulin production and complement activation (Extended Data Fig. 7d,e and Supplementary Tables 4 and 5–12). In the cohort with brain fog, there were changes in genes associated with vitamin A metabolism (for example, *DGAT2*, *DHRS9*) and regulation of leukocyte homeostasis (for example, *CXCL10*, *IL6R*) (Extended Data Fig. 7f and Supplementary Tables 4 and 5–12).

**Fig. 6:**
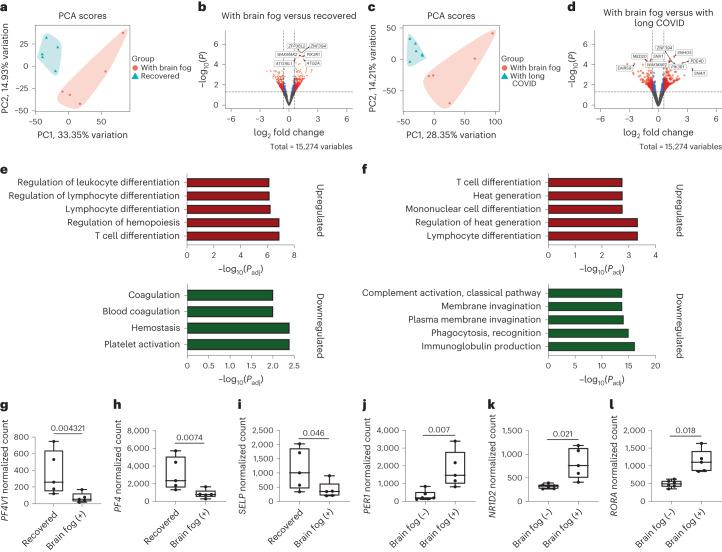
Immunovascular dysfunction in long COVID blood samples. **a**, PCA plot of brain fog versus recovered PBMC samples. **b**, Volcano plot depicting DEGs (red circles) with a log_2_ fold change > 0.58 or < −0.58 (vertical dashed lines) and *P* < 0.05 (horizontal dashed line). All DEGs with log_2_ fold change < 0.58 or > −0.58 and *P* < 0.05 are also displayed (blue circles). Data were analyzed using a Wald test with multiple comparisons controlled with an FDR. **c**, PCA plot of brain fog versus recovered PBMC samples. **d**, Volcano plot of DEGs (red circles) with a log_2_ fold change > 0.58 or < −0.58 (vertical dashed lines) and *P* < 0.05 (horizontal dashed line). DEGs with a log_2_ fold change < 0.58 or or > −0.58 and *P* < 0.05 are also displayed (blue circles). Data were analyzed using a Wald test with multiple comparisons controlled with an FDR. **e**,**f**, Top five upregulated and downregulated terms from brain fog versus recovered (**e**) and brain fog versus long COVID (**f**) cohorts. **g**–**i**, Normalized counts of *PF4V1* (**g**), *PF4* (**h**) and *SELP* (**i**) in brain fog versus recovered cohorts (*n* = 5 recovered, *n* = 5 with brain fog). **j**–**l**, Normalized counts of *PER1* (**j**), *NR1D2* (**k**) and *RORA* (**l**) in the cohort with brain fog versus the cohort with long COVID (*n* = 6 without brain fog (−), *n* = 5 with brain fog (+)). Data were analyzed using a Wald test with multiple comparisons controlled with an FDR. The box plots display the minimum and maximum values (whiskers), the median (solid line) and the IQR (upper and lower box) with significance set at *P* < 0.05. Statistical significance was assessed using DESeq2 with a Wald test and Benjamini–Hochberg correction. Source data

When comparing the cohort with brain fog to the recovered and long COVID cohorts, there were 1,156 and 1,078 DEGs, respectively (Fig. 6a–d). Principal component analysis (PCA) plots showed a clear separation of the cohort with brain fog from the recovered and long COVID cohorts (Fig. 6a–d). Compared to the recovered cases, there was a strong enrichment in upregulated terms for pathways related to T cell differentiation and activation (for example, PRDM1, TNF), TGFβ signaling (for example, *SMAD3*, *SNAI1*, *SMURF1*) and regulation of angiogenesis (for example, *HES1*, *DLL1*, *HIF1A*), while there was downregulation in genes involved in platelet activation, signaling and aggregation (for example, *PF4V1*, *PF4*, *TREML1*) and hemostasis (for example, *F13A1*, *GP1BA*, *GP1BB*) (Fig. 6e and Supplementary Tables 4 and 5–12). We also compared the transcriptome profile of individuals with and without brain fog in our cohort with long COVID. Upregulated genes were enriched in pathways related to T cell differentiation and activation (for example, *RUNX3*, *IFNG*, *TNFSF9*), negative regulation of the immune response (for example, *WASL*, *ID2*, *TNFAIP3*) and circadian regulation of gene expression (for example, *RORA*, *PER1*, *NRIP1*) (Fig. 6f,j–l). Pathways related to immunoglobulin, production, defense responses and B cell activation were among those downregulated, including immunoglobulin production (*IGKV1–12*, *IGKV1–17*), adaptive immune response (for example, *CX3CR1*, *FCGR1BP*) and B cell activation (*HDAC9*, *CD180*, *MNDA*) (Extended Data Fig. 7 and Supplementary Tables 4 and 5–12). In agreement with previous studies, several factors involved in the coagulation pathway were downregulated specifically in the cohort with brain fog, including *PF4*, *PF4V1* and *SELP* (Fig. 6g–i)^32^.

**Extended Data Fig. 8:**
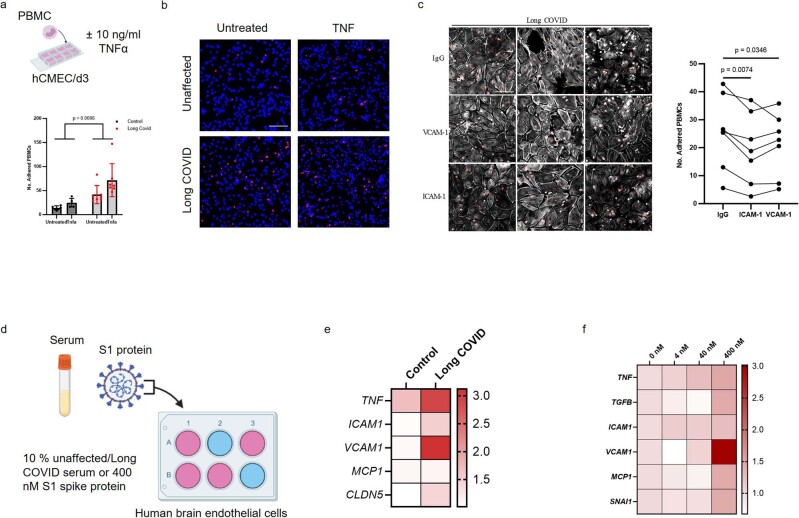
Adhesion of Long COVID PBMCs to human brain endothelial cells. **a**, **b**) Peripheral blood mononuclear cells (PBMCs) from Long COVID participants show greater adherence to the human brain endothelial cell line, hCMEC/d3, in the presence or absence of 10 ng/ml TNFa (n = 5 Control, n = 7 Long COVID). Error bars are mean ± s.d. Data in a was analysed by two-way ANOVA with Tukey correction. Each datapoint represents one patient. **c**) PBMCs from long COVID patients in the presence of IgG, VCAM-1 or ICAM-1 blocking antibodies (n = 6 long COVID patients). Data was analysed by repeated measures one-way ANOVA with Tukey correction for multiple comparisons. Each datapoint represents one patient. **d**) Schematic of experiments to assess the effect of long COVID patient serum or spike protein on human brain endothelial cells. **e**) Exposure of hCMEC/d3 cells to 10 % serum from healthy or long COVID participants and quantification of gene expression changes by q-RT-PCR. Long COVID serum significantly increased *TNF* (*P* = 0.0006) and *VCAM1* (*P* < 0.0001) vs control serum. **f**) Exposure of hCMEC/d3 cells to vehicle or 4, 40 or 400 nM S1 spike protein and quantification of gene expression changes by q-RT-PCR. 400 nM S1 spike protein significantly increased *TNF* (*P* = 0.024), *TGFB1* (*P* = 0.0274), *VCAM1* (*P* < 0.0001), *MCP1* (*P* = 0.0269) and *SNAI1* (*P* = 0.0389) vs vehicle. For e, n = 4 healthy serum samples and n = 8 long COVID serum samples were used. Scale bar – 50 µm. Schematics in a and d created with BioRender.com. Source data

We next examined immunovascular interactions in PBMCs isolated from patients with COVID-19 and found increased adhesion of PBMCs to human brain endothelial cells in the cohort with long COVID compared to unaffected individuals, which was heightened in the presence of TNF and only modestly affected by blocking antibodies against ICAM-1 and VCAM-1 (Extended Data Fig. 8a,b). Furthermore, exposure of human brain endothelial cells to 10% serum from recovered individuals and individuals with long COVID resulted in the upregulation of *ICAM1*, *VCAM1* and *TNF* transcripts compared to sera from unaffected individuals (Extended Data Fig. 8c,d). Previous studies indicated a role for S protein persistence in coagulation dysregulation and brain injury, so we explored how S protein affects endothelial cell activity. Exposure of human brain endothelial cells to S1 protein led to a dose-dependent increase in *TNF*, *TGFβ*, *ICAM1* and *VCAM1* mRNA (Extended Data Fig. 8e) after 72-h treatment with 0–400 nM S1 spike protein.

## Discussion

Our results suggest that long COVID-derived brain fog is associated with BBB disruption and sustained systemic inflammation. BBB dysfunction was unique to the cohort with brain fog, with disruption evident up to 1 year after active infection in multiple neuroanatomical regions, including the TLs and frontal cortex. BBB dysfunction was not apparent in patients with anosmia without accompanying brain fog implying this might not be a major driver of this symptom. Instead, accumulation of infiltrating T cells expressing IFNγ depleted sensory neurons in patients with anosmia^33^. Inflammation in the olfactory epithelium may result in cerebrovascular damage; higher-resolution MRI may help to tease apart these changes. We observed a significant correlation between BBB disruption in the TLs with the duration of anosmia. BBB disruption in the TL may be linked to anosmia because it contains important regions that form part of the primary olfactory cortex, including the piriform cortex, amygdala and entorhinal cortex, with direct connections to and from the olfactory bulb^33–35^. Our transcriptome data also point to aberrant T cell activation and regulation of IFN production. Previous work revealed an upregulation of activated T cells up to 8 months after infection^36^. T cell activation and waning of the innate and humoral immune response in patients recovering from COVID-19 has also been observed^37^. Dysregulation of the coagulation system has been strongly suggested as a key driver of long COVID and this was the most significantly dysregulated pathway in our cohort with brain fog^32,38^.

Structurally, there was reduced brain and WM volume in individuals with brain fog and recovered patients, suggesting that these changes do not primarily drive the fatigue and cognitive impairment associated with brain fog. Similar findings have been reported by others, including longitudinal changes in brain volume and cortical thinning after mild SARS-CoV-2 infection^39^. Neuroimaging has been used to detect other cerebrovascular changes in the brain after SARS-CoV-2 infection, including cerebral microbleeds, hypometabolism and cerebral hypoperfusion^40–44^.

BBB dysfunction was associated with neurological impairment during the active phase of SARS-CoV-2 infection, with increased serum levels of the astrocytic protein S100β together with increased levels of IL-6, bFGF and IL-13 suggesting that a heightened systemic inflammatory response may drive BBB dysfunction. Serum levels of S100β are elevated in several neurological disorders including epilepsy, traumatic brain injury and schizophrenia^45–47^. BBB dysfunction also increases with aging, which is an important risk factor for COVID severity^48,49^. S100β was associated with age in our acute cohort, which may explain the differences observed in those with brain fog; however, controlling for age still revealed a strong association with acute brain fog. Importantly, participant age was not associated with BBB permeability in our cohort with long COVID, suggesting that BBB disruption is more probably due to the neurological symptoms of long COVID. BBB dysfunction correlated with changes in brain volume and cortical thickness, most notably reduced GBV and increased CSF volume. Similar associations have been reported in bipolar disorder and systemic lupus erythematosus, where individuals with severe BBB disruption had more extensive brain volume loss or greater psychiatric morbidity^50,51^. This implies that changes in BBB function are closely related to changes in brain structure and ultimately function. However, longitudinal studies are needed to determine if BBB disruption during acute infection predisposes to the development of long COVID-associated brain fog.

Patients with long COVID had elevated levels of IL-8, GFAP and TGFβ, with TGFβ specifically increased in the cohort with brain fog. GFAP is a robust marker of cerebrovascular damage and is elevated after repetitive head trauma, reflecting BBB disruption, as seen in contact sport athletes and in individuals with self-reported neurological symptoms in long COVID^26,52,53^. Interestingly, TGFβ was strongly associated with BBB disruption and structural brain changes. TGFβ has been implicated in the pathogenesis of chronic fatigue syndrome, a condition with clinical similarities to long COVID^54–56^.

Insights from animal models and postmortem tissue examined the impact of acute infection on BBB integrity. Brain sections from patients who died from COVID-19 showed fibrinogen extravasation and coagulation system dysregulation^21,22^, while mouse models revealed changes in blood vessel morphology with the appearance of string vessels, that is, pathological ‘ghost’ vessels without endothelial cells^28^. Biomarker studies in patients convalescing from COVID-19 also consistently highlighted the involvement of inflammation and coagulation system dysregulation^32,38^.

Persistence of viral components, such as S protein, has been hypothesized to be responsible for long COVID-associated neurological symptoms^57–59^. S protein persistence may be involved in neurological sequelae as direct brain injection was associated with coagulation dysregulation and neurodegeneration. This suggests that S protein may have a long half-life in the body. In support of this, immune cells were identified with S protein up to 15 months after infection^60^. Furthermore, we showed that exposure of brain endothelial cells to S protein resulted in an activated endothelial cell phenotype with upregulation of inflammatory cytokines and cell adhesion molecules and probably has a role in long COVID-associated brain fog. Reinforcing these findings, previous studies showed that S protein promoted tight junction degradation, endothelial cell activation and increased adherence of immune cells^23,61^. The long-lasting influence of S protein on cerebrovascular function is unknown and should be investigated in future studies, especially considering the longevity of brain endothelial cells.

Long COVID is a substantial burden in many patients after recovery from COVID-19. Patients describe fatigue, memory loss and dyspnea as some of the key symptoms of long COVID, while another subset of patients describe ‘brain fog’ like the one commonly reported in postconcussive syndrome and chronic fatigue syndrome^62,63^. Our data suggest that BBB disruption occurs during acute infection and long COVID, where it is strongly associated with cognitive impairment. Our work provides objective evidence for a link between BBB disruption and cognitive impairment within a cohort of patients with long COVID. Further longitudinal studies are required to examine changes in BBB permeability over time and in other postviral illnesses; however, targeted regulation of BBB integrity could now potentially be considered for the treatment of patients with brain fog associated with long COVID.

Our study has some limitations. First, we did not have access to CSF samples from our cohort to confirm molecular BBB breakdown in those with brain fog. However, other studies found increased CSF permeability and BBB disruption in a subset of patients infected with SARS-CoV-2 with elevated Q-albumin ratios that correlated with markers of inflammation^64^. Patients with severe neurological complications from SARS-CoV-2 infection have an increased CSF Q-albumin ratio indicating BBB disruption, coupled with elevated CSF proteins, such as IL-8, which are associated with BBB disruption^65^. Blood–CSF barrier breakdown was the most frequent pathological finding in a multicenter study of 127 patients with COVID-19 with neurological impairment^66^.

Second, we did not examine longitudinal changes in BBB function in our cohort with long COVID; it will be important to determine how long it takes for individuals to recover and if there is resolution of BBB function and prolonged inflammation. As many as half of those infected with SARS-CoV-2 reported no, or incomplete, recovery between 6 and 18 months after infection with 11% reporting deterioration in symptoms^67^. Understanding the long-term outcome of long COVID will be critical to develop treatment options for this large group of individuals.

Our study is also limited by a small sample size. Future studies with larger patient cohorts should perform unbiased proteome profiling on blood and CSF samples. In agreement with our study, another study also found elevated levels of markers of neurological injury and BBB disruption, such as GFAP, in individuals with long COVID with self-reported neurological symptoms^53^. Ultimately, expanding the use of clinical tools focused on understanding the role of the BBB in postviral illnesses may lead to better treatment and management strategies for patients in the future.

## Methods

### Study participants

Participants included patients who had recovered from COVID-19, male or female aged 18 and above with and without neurological symptoms. Participants with long COVID, with symptom persistence over 12 weeks from infection, were also recruited. Candidates were excluded if they had a history of a neurological disorder that may better explain the results of the study such as epilepsy, brain trauma, neuropsychiatric disorder or mild cognitive impairment. Suitable candidates proceeded to assessment with DCE-MRI imaging, Q-SIT olfactory testing and a review of pulmonary imaging and hematological parameters at the time of the COVID-19 diagnosis. The Joint Research Ethics Committee of St James’s and Tallaght Hospital’s approved the study and written informed consent was obtained from all participants. Research was performed according to the principles of the Declaration of Helsinki of 2013. The legal basis for the study was consent according to General Data Protection Regulation principles.

### Olfactory testing

The olfactory function of participants was assessed using the Q-SIT. The Q-SIT is a standardized and validated three-item odor identification screen^68^. A score of 2 or more is a normal test and a cutoff score of 1 or less is an abnormal test for anosmia. Q-SIT has displayed high positive and negative predictive value in detecting olfactory dysfunction in patients with COVID-19. In addition, the Q-SIT is a tear-off card test that is disposable, so there is no concern about contamination and transmission of disease from patients with COVID-19 (ref. ^69^).

### DCE-MRI

BBB permeability maps were created using the slope of contrast agent concentration in each voxel over time, calculated using a linear fit model as described previously^70–72^. Thresholds of high permeability were defined by the 95th percentile of all slopes in a previously examined control group. Imaging was performed with a 3T Philips Achieva scanner. Sequences included a T1-weighted anatomical scan (3D gradient echo; time to echo (TE)/repetition time (TR) = 3 ms/6.7 ms; acquisition matrix 268 × 266; voxel size: 0.83 mm × 0.83 mm × 9 mm), T2-weighted imaging (TE/TR = 80 ms/3,000 ms; voxel size: 0.45 mm × 0.45 mm × 4 mm), fluid-attenuated inversion recovery (TE/TR = 125 ms/11,000 ms; voxel size: 0.45 mm × 0.45 mm × 4 mm). For the calculation of precontrast longitudinal relaxation time (T10), the variable flip angle method was used (3D T1W-FFE; TE/TR = 2.78 ms/5.67 ms; acquisition matrix: 240 × 184; voxel size: 0.68 mm × 0.68 mm × 5 mm; flip angles: 10°, 15°, 20°, 25° and 30°). The DCE sequence was then acquired (axial, 3D T1w-FFE; TE/TR = 2.78 ms/5.6 ms; acquisition matrix: 240 × 184; voxel size: 0.68 mm × 0.68 mm × 5 mm; flip angle: 20°; Δ*t* = 22.2 s; temporal repetitions: 61; total scan length: 22.6 min). An intravenous bolus injection of the contrast agent gadobenate dimeglumine (Bracco Diagnostics Inc.) was administered using an automatic injector after the first three DCE repetitions. To control for interindividual variabilities due to heart rate, blood flow or rate of contrast injection, each voxel’s leakage rate was normalized to that of the superior sagittal sinus. Each of the 61 temporal repetitions was manually inspected for movement artifacts and was manually excluded from the DCE analysis protocol. If movement artifacts were detected on more than 10% of the entire scan, then the individual was excluded from the analysis. No patients were excluded from the DCE-MRI analysis in this study. One patient from the cohort with long COVID underwent T1 imaging only. Quantification of BBB dysfunction was calculated as described previously^70,72^. Briefly, image preprocessing involved image segmentation, registration and normalization to Montreal Neurological Institute (MNI) space using SPM12. To calculate the slow accumulation of contrast agent, a linear fit was applied to min 6–22 of the concentration curve of each voxel with normalization to the venous input function, which is the leakage rate of the superior sagittal sinus. The percentage of the suprathreshold voxels was used as a measure reflecting global BBB leakage.

### Volumetric and thickness measurements

T1-weighted anatomical images were uploaded to the volBrain online brain volumetry software (https://www.volbrain.net/)^73^ and analyzed with vol2brain 1.0, which is an online pipeline that registers images to the MNI space and reports the volumes of expert-labeled anatomical structures as a percentage of the TIV. We analyzed the volume of the right and left cerebral and cerebellar GM/WM, FLs, TLs, OLs, parietal lobes and CSF along with the thickness of the FLs, TLs, OLs and parietal lobes. All volume data was normalized to the TIV, which is the sum of GM, WM and CSF. Volumes were expressed as a percentage of the TIV. Sixty age-matched and sex-matched healthy control scans were randomly selected from the IXI dataset (https://brain-development.org/ixi-dataset/), which represents 10% of the entire dataset. All scans were performed on the same Philips 3T system at Hammersmith Hospital. Volumetric maps for comparisons between COVID^+^ and COVID^−^ groups were generated in xjView after automatic brain segmentation in the CAT12 toolbox with default parameters and subsequent smoothing with an 8-mm kernel. Thickness maps for comparisons between COVID^+^ and COVID^−^ groups were generated in the CAT12 toolbox run in SPM12 in MATLAB R2021a after brain segmentation, as above, and smoothing with a 15-mm kernel. A two-sample *t*-test was used for statistical analysis with age, sex and TIV as covariates.

### Sample collection

Blood samples were collected into serum separator tubes and EDTA-coated tubes for serum and PBMC isolation, respectively. Serum was separated by centrifugation at 800*g* for 10 min at room temperature. PBMCs were separated by layering the blood samples diluted twofold in PBS (cat. no. 14190, Thermo Fisher Scientific) over a Lymphoprep density gradient medium (cat. no. 07851, STEMCELL Technologies) followed by centrifugation at 400*g* for 25 min at room temperature at 0 break and 0 acceleration. Plasma was collected and stored at −80 °C and the PBMC layer was collected into a new 50-ml Falcon tube, resuspended to 50 ml with PBS and centrifuged at 800*g* for 5 min at room temperature. PBMCs were resuspended in 50 ml PBS and centrifuged at 400*g* for 10 min at room temperature. PBMCs were resuspended to 2 × 10^6^ cells per ml in Roswell Park Memorial Institute (RPMI) 1640 medium with l-glutamine (cat. no. LZBE12-702F, Lonza) supplemented with 50% FCS (cat. no. F7524, Merck) and 10% dimethylsulfoxide (cat. no. D5879, Merck) and frozen at −80 °C overnight before being moved to liquid nitrogen.

### Multiplex assays

A 10-plex Luminex assay (cat. no. LXSAHM-10, R&D Systems) was used for cytokine profiling. Serum samples were diluted twofold in sample dilution buffer. Then, 50 µl of sample or standard was pipetted in duplicate into each wall of an assay 96-well plate. Then, 50 µl of diluted Microparticle Cocktail was added to each well, the plate was covered and incubated for 2 h at room temperature on a shaker at 300 r.p.m. Wells were washed three times with wash buffer before addition of 50 µl of diluted Biotin-Antibody Cocktail. The plate was covered and incubated for 1 h at room temperature on a shaker at 300 r.p.m. Wells were washed as above before the addition of 50 µl of diluted streptavidin-phycoerythrin to each well. The plate was covered and incubated for 30 min at room temperature on a shaker at 300 r.p.m. Wells were washed as above before microparticles were resuspended in 100 µl of wash buffer. The plate was incubated for 2 min at room temperature on a shaker at 300 r.p.m. and was read on a MAGPIX plate reader with the xPONENT software (Luminex). A Bio-Plex Pro Human Cytokine 27-plex Assay (cat. no. M500KCAF0Y, Bio-Rad Laboratories) was used for cytokine profiling. Samples and standards were diluted fourfold in sample dilution and plates were processed according to the manufacturer’s instructions. A ProcartaPlex Human Coagulation Panel 3 4-Plex (cat. no. EPX040-10825-901, Thermo Fisher Scientific) was used for coagulation factor profiling. Plasma samples were collected in citrate tubes and spun at 2,000*g* for 10 min. Plasma was diluted 1:500 and assayed according to the manufacturer’s instructions. Separate enzyme-linked immunosorbent assays (ELISAs) were used for additional inflammatory, coagulation and BBB markers and included human tissue factor (1:10 dilution, cat. no. HUFI00258, AssayGenie), human D-dimer (1:20,000 dilution, cat. no. EHDDIMER, Thermo Fisher Scientific), human plasminogen activator inhibitor-1 (1:200 dilution, cat. no. DY9387-05, Bio-Techne), human UCH-L1 (1:2 dilution, cat. no. DY6007-05, Bio-Techne), human VCAM-1 (1:2,000 dilution, cat. no. DY809, Bio-Techne), human ICAM-1 (1:500 dilution, cat. no. DY720-05, Bio-Techne), human ECM-1 (1:1,000 dilution, cat. no. DY3937-05, Bio-Techne), human VEGF (1:5 dilution, cat. no. DY293B, Bio-Techne) and human BDNF (1:5 dilution, cat. no. DY248, Bio-Techne). For analytes at the lower limit, the lower limit of detection was used.

### Dot blot

Plasma samples were spotted (2 µl) onto a 0.2-µm nitrocellulose membrane (cat. no. 10401391, Whatman) and allowed to dry for 30 min. Membranes were blocked in 5% BSA (cat. no. A7906, Merck) in PBS with 0.1% Tween 20 (PBST) for 1 h at room temperature. Membranes were incubated overnight in primary antibody in blocking buffer. Membranes were washed three times for 5 min each in PBST, followed by incubation in secondary horseradish peroxidase (HRP)-conjugated antibodies. Membranes were washed three times for 5 min each in PBST and incubated with strong ECL substrate (cat. no. K-12045-D50, Advansta) for 2 min before being developed on a C-Digit (LI-COR Biosciences). Protein bands were quantified in ImageJ (National Institutes of Health). Primary antibodies used were mouse anti-GFAP (1:500 dilution, cat. no. G3893, Merck), rabbit anti-TGFβ (1:500 dilution, cat. no. ab92486, Abcam) and mouse anti-Phospho-Tau (1:500 dilution, cat. no. 10599853, Thermo Fisher Scientific). Secondary antibodies used were anti-mouse HRP (1:5,000 dilution, cat. no. A4416, Merck) and anti-rabbit HRP (1:5,000 dilution, cat. no. A6154, Merck).

### Quantitative PCR with reverse transcription

RNA was isolated from PBMCs and the human brain endothelial cell line hCMEC/d3 (cat. no. SCC066, Merck Millipore) with the Omega RNA Isolation Kit (cat. no. R6834-02, VWR) according to the manufacturer’s instructions. Complementary DNA was reverse-transcribed from 500 ng RNA with the High Capacity cDNA Reverse Transcription Kit (cat. no. 4368814, Applied Biosystems). Transcript levels were quantified on a StepOne Plus instrument (Applied Biosystems) with FastStart Universal SYBR Green Master (Rox) master mix (cat. no. 04913914001, Roche). Quantitative PCR with reverse transcription was performed with the following conditions: 95 °C × 2 min (95 °C × 5 s, 60 °C × 30 s) ×40, 95 °C × 15 s, 60 °C × 1 min, 95 °C × 15 s, 60 °C × 15 s. Relative gene expression levels were quantified using the comparative CT method (ΔΔ^Ct^). The expression levels of target genes were normalized to β-actin.

### Adhesion assay

hCMEC/d3 cells were cultured in EGM2-MV growth medium (cat. no. CC-3202, Lonza) and were stimulated with 10 ng ml^−1^ recombinant human TNF (cat. no. 300-01A, PeproTech) for 4 h in the presence or absence of 1 µg ml^−1^ anti-ICAM-1, anti-VCAM-1 or anti-PECAM-1 antibodies before incubation with 1 × 10^5^ MitoTracker Orange-labeled PBMCs (cat. no. M7510, Thermo Fisher Scientific) for 1 h at 37 °C. Cells were washed three times in PBS to remove unbound PBMCs and fixed in 4% formaldehyde (cat. no. F1635, Merck) for 10 min at room temperature. The number of adhered PBMCs was counted with the ImageJ cell counter plugin. Images were imported and converted to 8-bit and thresholded. Noise was removed with the despeckle function and images were converted to binary. The cell counter plugin was then used to count adhered PBMCs. Counts were averaged from five images per treatment.

### Serum and S protein treatment

hCMEC/d3 cells were seeded in 12-well plates at 2 × 10^5^ cells per well and grown to confluence. Medium was replaced with medium containing 10% serum from individuals with COVID and unaffected controls and incubated for up to 72 h. This was followed by RNA isolation. hCMEC/d3 cells were cultured in 12-well plates as described above and stimulated with 4, 40 and 400 nM Recombinant SARS-CoV-2 Spike S1 Subunit Biotin Protein (cat. no. BT10569, R&D Systems) for up to 72 h. RNA was isolated as described above.

### RNA-seq

PBMCs were thawed in a bead bath at 37 °C, topped up with RPMI medium to 10 ml and centrifuged at 300*g* for 5 min at room temperature. PBMCs were allowed to recover for 1 h at 37 °C in RPMI medium supplemented with 10% FCS before RNA isolation. RNA was isolated from 1 × 10^6^ PBMCs with the E.Z.N.A. Total RNA Kit I (Omega Bio-tek) according to the manufacturer’s recommendations. RNA samples were analyzed on a TapeStation 2200 (Agilent Technologies) and samples with an RNA integrity number value greater than 7 and ribosomal RNA ratio greater than 1 were used for library preparation and RNA-seq; 10 ng RNA was used for the SMART-Seq v4 Ultra Low Input RNA Kit for sequencing with the Nextera XT DNA Library Preparation Kit. RNA-seq with 100 bp paired-end reads and more than 20 million reads per sample was performed on a NovaSeq 6000 (Illumina). Raw FASTQ files were trimmed with Cutadapt (part of Trim Galore) and aligned to the Gencode GRCh38 Release 43 reference using STAR. The resulting BAM files were sorted with STAR and indexed with Samtools. Gene quantification was performed using the RSEM’s rsem-calculate-expression tool. Count matrices produced by RSEM were processed with tximport and differential expression analysis was performed using DESeq2 v.1.38.3 in R. Alignment metrics were generated using the Genome Analysis Toolkit (GATK)’s CollectAlignmentSummaryMetrics. The distribution of the bases within the transcripts was determined using GATK’s CollectRnaSeqMetrics. Quality control reports were generated with FastQC (part of Trim Galore) and amalgamated into a single report with MultiQC. GO was performed with the clusterProfiler package. Data were filtered for *P* < 0.05. The enrichGO function of clusterProfiler was then used with Benjamini–Hochberg adjustment and a cutoff of *P* < 0.05. GO enrichment was performed by selecting the biological processes subontology.

### Ethical approval

Informed consent was obtained from each participant. All ethical approvals were in place before the initiation of studies on humans. All experiments conformed to the principles set out in the World Medical Association Declaration of Helsinki and the Department of Health and Human Services Belmont Report. The St James’ Hospital ethics committee approved these studies.

### Statistical analysis

No statistical methods were used to predetermine sample sizes. SPSS v.28 (IBM Corporation) and Prism v.9 (GraphPad Software) were used for the statistical analysis. Prism v.9 was used to generate the graphs. Categorical variables were compared between groups with chi-squared tests. Cytokine data were log-transformed and analyzed with a general linear model, with age, sex and all comorbidities as covariates. For structural MRI analysis, a multivariate general linear model was used, with age, sex and TIV as covariates. Correlations were assessed with Pearson or Spearman rho correlation tests using partial correlations to control for age, sex and TIV. For repeated blood samples, matched samples were compared using a Wilcoxon signed-rank test. To control for multiple comparisons in multiplex assays, brain region MRI analysis and correlation analysis, FDR was applied using the Benjamini–Hochberg correction. A *P* < 0.05 was considered statistically significant. All quantitative PCR, ELISA and adhesion assays were performed in duplicate.

### Reporting summary

Further information on research design is available in the Nature Portfolio Reporting Summary linked to this article.

## Online content

Any methods, additional references, Nature Portfolio reporting summaries, source data, extended data, supplementary information, acknowledgements, peer review information; details of author contributions and competing interests; and statements of data and code availability are available at 10.1038/s41593-024-01576-9.

### Supplementary information

Supplementary Information Supplementary Tables 1–4.

Reporting Summary

Supplementary Tables 5–13 GO biological analysis of long COVID PBMCs.

### Source data

Source Data Fig. 1 Statistical source data.

Source Data Fig. 2 Statistical source data.

Source Data Fig. 3 Statistical source data.

Source Data Fig. 4 Statistical source data.

Source Data Fig. 5 Statistical source data.

Source Data Fig. 6 Statistical source data.

Source Data Extended Data Fig. 1 Statistical source data.

Source Data Extended Data Fig. 3 Statistical source data.

Source Data Extended Data Fig. 4 Statistical source data.

Source Data Extended Data Fig. 5 Statistical source data.

Source Data Extended Data Fig. 6 Statistical source data.

Source Data Extended Data Fig. 8 Statistical source data.

## Data Availability

Data supporting the findings of this study are available from the corresponding authors upon reasonable request. The RNA-seq data have been deposited at the NCBI’s Gene Expression Omnibus and are accessible under accession no. GSE251849. Publicly available MRI datasets can be accessed at https://brain-development.org/ixi-dataset/. This study did not generate new or unique reagents. Source data are provided with this paper.

## Code availability

No custom software was developed during this study. Open-source packages and libraries and corresponding versions used during the computational analysis are described in the Methods and Reporting Summary. MATLAB scripts and notebooks are available upon reasonable request from the corresponding authors.
