# Author Correction: Blood–brain barrier disruption and sustained systemic inflammation in individuals with long COVID-associated cognitive impairment

**DOI:** 10.1038/s41593-024-01644-0

**Published:** 2024-04-16

**Authors:** Chris Greene, Ruairi Connolly, Declan Brennan, Aoife Laffan, Eoin O’Keeffe, Lilia Zaporojan, Jeffrey O’Callaghan, Bennett Thomson, Emma Connolly, Ruth Argue, James F. M. Meaney, Ignacio Martin-Loeches, Aideen Long, Cliona Ni Cheallaigh, Niall Conlon, Colin P. Doherty, Matthew Campbell

**Affiliations:** 1 Smurfit Institute of Genetics, Trinity College Dublin, Dublin, Ireland; 2 Department of Neurology, Health Care Centre, St James’s Hospital, Dublin, Ireland; 3 The Irish Longitudinal Study on Ageing, School of Medicine, Trinity College Dublin, Dublin, Ireland; 4 Clinical Research Facility, St James’s Hospital, Dublin, Ireland; 5 Thomas Mitchell Centre for Advanced Medical Imaging (CAMI), St. James’s Hospital & Trinity College Dublin, Dublin, Ireland; 6 Department of Intensive Care Medicine, Multidisciplinary Intensive Care Research Organization, Trinity Centre for Health Sciences, St James’s University Hospital, Dublin, Ireland; 7 Trinity Translational Medicine Institute, Trinity College Dublin, St James’s Hospital, Dublin, Ireland; 8 Department of Immunology, St James’s Hospital, Dublin, Ireland; 9 St James’s Hospital, Tallaght University Hospital, Trinity College Dublin Allied Researchers (STTAR) Bioresource, Trinity College Dublin, Dublin, Ireland; 10 Academic Unit of Neurology, Biomedical Sciences Institute, Trinity College Dublin, Dublin, Ireland; 11 FutureNeuro, Science Foundation Ireland Research Centre for Chronic and Rare Neurological Diseases, Royal College of Surgeons in Ireland, University of Medicine and Health Sciences, Dublin, Ireland

**Keywords:** Neuro-vascular interactions, Neuroimmunology, Diseases of the nervous system

Correction to: *Nature Neuroscience* 10.1038/s41593-024-01576-9, published online 22 February 2024.

In the version of the article initially published, James F. M. Meaney (Thomas Mitchell Centre for Advanced Medical Imaging (CAMI), St. James’s Hospital & Trinity College Dublin, Dublin, Ireland) was mistakenly omitted from the author list and contributions (diagnostic reporting of MRI scans) and has now been added in the HTML and PDF versions of the article.

