# Supplementary material for: Blood–brain barrier disruption and sustained systemic inflammation in individuals with long COVID-associated cognitive impairment
Source: Nat Neurosci. 2024 Feb 22;27(3):421–32. doi: 10.1038/s41593-024-01576-9 (PMC10917679; doi:10.1038/s41593-024-01576-9)
Supplement: Supplementary file 1 — Supplementary Tables 1–4. [file 41593_2024_1576_MOESM1_ESM.pdf]

nature neuroscience

Article https://doi.org/10.1038/s41593-024-01576-9

Blood–brain barrier disruption and sustained systemic inflammation in individuals with long COVID-associated cognitive impairment

In the format provided by the authors and unedited

Supplementary Table 1. Demographics of acute COVID cohort. Data was analysed by two-sided Kruskal-Wallis test with Bonferroni correction for multiple comparisons or two-sided z-test for comparisons of column proportions.

	Unaffected	Mild	Moderate	Severe	p-value
Gender (M/F)	11/14	8/35	2/8	12/11	
Age (years)	44	40	42	54	0.012
Hospitalisation					
Hospitalisation		0 (0%)	8 (80%)	21 (91%)	
No hospitalisation		43 (100%)	2 (20%)	2 (9%)	
ICU					
Required		0 (0%)	0 (0%)	2 (9%)	
Not required		43 (100%)	10 (100%)	21 (91%)	
Oxygen					
Required		0 (0%)	0 (0%)	17 (74%)	
Not required		43 (100%)	10 (100%)	6 (26%)	
COVID medication					
Required		0 (0%)	4 (40%)	21 (91%)	
Not required		43 (100%)	6 (60%)	2 (9%)	
			Brain Fog (-)	Brain Fog (+)	p-value
Gender (M/F)			18/44	4/10	
Age (years)			43	54	0.011
Hospitalisation					0.026
Hospitalisation			42 (68%)	5 (36%)	
No hospitalisation			20 (32%)	9 (64%)	
ICU					
Required			2 (3%)	0 (0%)	
Not required			60 (97%)	14 (100%)	
Oxygen					0.006
Required			10 (16%)	7 (50%)	
Not required			52 (84%)	7 (50%)	
COVID medication					0.033
Required			17 (27%)	8 (57%)	
Not required			45 (73%)	6 (43%)	

Supplementary Table 2. Demographics of Long COVID cohort. Data was analysed by two-sided Kruskal-Wallis test with Bonferroni correction for multiple comparisons.

	Unaffected	Recovered	Brain Fog (-)	Brain Fog (+)	p-value
Gender (M/F)	18/42	4/6	2/9	0/11	
Age	40	40	38	46	
Comorbidity score		0.3	0.36	0.33	
Time from PCR+ to scan		46.6	170.6	211	0.0004
Anosmia, n (%)		0 (0%)	8 (73%)	8 (73%)	
Anosmia duration (days)		0	110	121	0.002
Q-SIT		2.6	1.5	1.4	0.009
MOCA				24.9	

Supplementary Table 3. Volumetric measurements and statistical analysis. Data was analysed by ANCOVA adjusting for age and sex with Bonferroni correction for multiple comparisons.

	No adjustment	Age adjusted	FDR corrected

Omnibus statistics

Region	F	P-value	eta	F	P-value	eta	q-value	Discovery?
Global brain volume	5.749	0.001	0.167	5.092	0.003	0.152	0.001683	Yes
CSF volume	6.769	0.0004	0.191	6.251	0.0007	0.181	0.000583	Yes
White matter volume	11.794	<0.0001	0.291	11.883	<0.0001	0.295	0.000005	Yes
Grey matter volume	2.34	0.079	0.075	2.974	0.036	0.095	0.01818	No
Cerebrum volume	6.281	0.0007	0.18	5.79	0.001	0.17	0.000721	Yes
Cerebrum right volume	6.276	0.0007	0.18	5.812	0.001	0.17	0.000721	Yes
Cerebrum left volume	6.083	0.0008	0.175	5.543	0.002	0.164	0.001188	Yes
Cerebrum white matter volume	11.016	<0.0001	0.278	11.126	<0.0001	0.282	0.000008	Yes
Cerebrum white matter right volume	11.328	<0.0001	0.283	11.45	<0.0001	0.288	0.000007	Yes
Cerebrum white matter left volume	10.54	<0.0001	0.269	10.636	<0.0001	0.273	0.000008	Yes
Cerebrum grey matter volume	1.981	0.123	0.065	2.367	0.076	0.077	0.030704	No
Cerebrum grey matter right volume	1.787	0.156	0.059	2.123	0.103	0.07	0.037154	No
Cerebrum grey matter left volume	2.168	0.098	0.07	2.587	0.058	0.084	0.024408	No
Frontal cortex mm	2.842	0.043	0.09	2.848	0.042	0.091	0.019741	No
Frontal cortex right mm	2.502	0.065	0.08	2.313	0.082	0.075	0.031854	No
Frontal cortex left mm	3.139	0.029	0.099	3.322	0.024	0.105	0.012758	No
Frontal pole mm	9.975	<0.0001	0.258	10.87	<0.0001	0.277	0.000008	Yes
Frontal pole right mm	5.225	0.002	0.154	5.342	0.002	0.159	0.001188	Yes
Frontal pole left mm	14.173	<0.0001	0.331	15.203	<0.0001	0.349	5.33E-07	Yes
Superior frontal gyrus mm	5.918	0.001	0.171	8.126	<0.0001	0.223	0.000091	Yes
Superior frontal gyrus right thickness	5.004	0.003	0.149	6.604	0.0005	0.189	0.000421	Yes
Superior frontal gyrus left thickness	6.542	0.0005	0.186	8.879	<0.0001	0.239	0.000051	Yes
Medial temporal gyrus mm	3.585	0.017	0.111	2.84	0.043	0.091	0.019741	No
Medial temporal gyrus right mm	2.981	0.036	0.094	2.269	0.086	0.074	0.03217	No
Medial temporal gyrus left mm	3.349	0.023	0.105	2.673	0.052	0.086	0.022835	No
Superior temporal gyrus mm	8.243	<0.0001	0.223	8.194	<0.0001	0.224	0.000091	Yes
Superior temporal gyrus right mm	7.579	0.0001	0.209	7.34	0.0002	0.206	0.000198	Yes
Superior temporal gyrus right mm	5.775	0.001	0.168	5.169	0.002	0.154	0.001188	Yes

Supplementary Table 4. Correlations between BBB disruption, structural brain data and clinical measurements. Data was analysed by ANCOVA adjusting for age and sex with Bonferroni correction for multiple comparisons.

Correlations between % BBB disruption, brain region and clinical data

Region	Age-uncorrected rho	Age-uncorrected p-value	Age-corrected rho	Age-corrected p-value
Global brain volume	-0.555	0.001	-0.614	0.00039
Cerebrum volume	-0.526	0.002	-0.57	0.001
Cerebrum right volume	-0.521	0.003	-0.544	0.002
Cerebrum left volume	-0.509	0.003	-0.557	0.002
Cerebrum white matter volume	-0.468	0.008	-0.505	0.005
Cerebrum white matter right volume	-0.449	0.011	-0.487	0.007
Cerebrum white matter left volume	-0.491	0.005	-0.516	0.004
Cerebrum grey matter volume	-0.293	0.11	-0.342	0.069

Cerebrum grey matter right volume	-0.29	0.113	-0.329	0.081
Cerebrum grey matter left volume	-0.307	0.093	-0.35	0.062
Cerebellum volume	-0.143	0.442	-0.195	0.312
Cerebellum white matter volume	-0.259	0.16	-0.316	0.095
Cerebellum grey matter volume	-0.002	0.99	-0.041	0.831
CSF volume	0.552	0.001	0.595	0.000664
Brainstem volume	-0.06	0.749	-0.103	0.596
Amygdala volume	0.433	0.015	0.389	0.037
Hippocampus volume	0.15	0.419	0.143	0.459
QSIT score	-0.428	0.218	-0.243	0.561
Anosmia duration	0.656	0.039	0.603	0.114
MOCA	-0.049	0.893	-0.08	0.851
