# Supplementary material for: Blood–brain barrier disruption and sustained systemic inflammation in individuals with long COVID-associated cognitive impairment
Source: Nat Neurosci. 2024 Feb 22;27(3):421–32. doi: 10.1038/s41593-024-01576-9 (PMC10917679; doi:10.1038/s41593-024-01576-9)
Supplement: Supplementary file 2 — Reporting Summary [file 41593_2024_1576_MOESM2_ESM.pdf]

nature portfolio

Corresponding author(s): Matthew Campbell

Last updated by author(s): Jan 8, 2024

Reporting Summary

Nature Portfolio wishes to improve the reproducibility of the work that we publish. This form provides structure for consistency and transparency in reporting. For further information on Nature Portfolio policies, see our Editorial Policies and the Editorial Policy Checklist.

Statistics

For all statistical analyses, confirm that the following items are present in the figure legend, table legend, main text, or Methods section.

n/a	Confirmed	
☐	☒	The exact sample size (*n*) for each experimental group/condition, given as a discrete number and unit of measurement
☐	☒	A statement on whether measurements were taken from distinct samples or whether the same sample was measured repeatedly
☐	☒	The statistical test(s) used AND whether they are one- or two-sided<br>*Only common tests should be described solely by name; describe more complex techniques in the Methods section.*
☐	☒	A description of all covariates tested
☐	☒	A description of any assumptions or corrections, such as tests of normality and adjustment for multiple comparisons
☐	☒	A full description of the statistical parameters including central tendency (e.g. means) or other basic estimates (e.g. regression coefficient) AND variation (e.g. standard deviation) or associated estimates of uncertainty (e.g. confidence intervals)
☐	☒	For null hypothesis testing, the test statistic (e.g. *F*, *t*, *r*) with confidence intervals, effect sizes, degrees of freedom and *P* value noted<br>*Give P values as exact values whenever suitable.*
☒	☐	For Bayesian analysis, information on the choice of priors and Markov chain Monte Carlo settings
☒	☐	For hierarchical and complex designs, identification of the appropriate level for tests and full reporting of outcomes
☐	☒	Estimates of effect sizes (e.g. Cohen's *d*, Pearson's *r*), indicating how they were calculated

Our web collection on statistics for biologists contains articles on many of the points above.

Software and code

Policy information about availability of computer code

Data collection	For RNA sequencing data, we used CutAdapt v4.3 to trim FASTQ files, STAR v2.7.11 for sequence alignment, SamTools v1.18 for indexing BAM files. Cytokine data was collected on a MAGPIX with xPONENT software v4.3. Microscopy data was collected on a Zeiss LSM 710 confocal microscope. q-RT-PCR data was collected on a StepOnePlus Real-Time PCR system with StepOnePlus software v2.3.
Data analysis	For RNA seq analysis, RSEM was used for gene quantification and DESeq2 v1.38.3 for differential expression analysis. EnhancedVolcanoPlot was used for generating volcano plots in R-4.3.1. clusterProfiler was used for gene ontology analysis. MATLAB 2021a was used to analyse dynamic contrast-enhanced images in SPM12. Volumetric and thickness measurements were performed with volbrain https://www.volbrain.net/ and volume/thickness maps were generated in CAT12 run in SPM12 https://neuro-jena.github.io/cat12-help/. Cytokine data was analysed in IBM SPSS Statistics version 28.0.1.1. GraphPad Prism v9.0 was used to analyse q-RT-PCR data. ImageJ v1.53 was used to analyse adhesion assays.

For manuscripts utilizing custom algorithms or software that are central to the research but not yet described in published literature, software must be made available to editors and reviewers. We strongly encourage code deposition in a community repository (e.g. GitHub). See the Nature Portfolio guidelines for submitting code & software for further information.

Data

Policy information about availability of data

All manuscripts must include a data availability statement. This statement should provide the following information, where applicable:

- Accession codes, unique identifiers, or web links for publicly available datasets
- A description of any restrictions on data availability
- For clinical datasets or third party data, please ensure that the statement adheres to our policy

Data supporting the findings of this study are available from the corresponding authors. Source data are provided with this paper. Neuroimaging data from healthy volunteers is available at https://brain-development.org/ixi-dataset/. Neuroimaging data from COVID participants is available from the corresponding author upon request. RNA-seq data is available on the NCBI GEO NGS repository under the accession GSE251849. Gencode GRCH38 Release was used for alignment.

Research involving human participants, their data, or biological material

Policy information about studies with human participants or human data. See also policy information about sex, gender (identity/presentation), and sexual orientation and race, ethnicity and racism.

Reporting on sex and gender	Participant sex was self-reported. We performed analysis and controlled for gender as a covariate in the analysis. Sex-specific analysis was not performed due to the low number of men in brain fog and Long COVID cohorts.
Reporting on race, ethnicity, or other socially relevant groupings	n/a
Population characteristics	SARS-CoV-2 infected patients >18 years old with PCR confirmed infection. Additional details are in Table 1<br>Recovered participants with PCR confirmed infection that had completely recovered from acute infection with no lingering symptoms.<br>Long COVID participants with PCR confirmed infection that recovered from acute infection with symptoms persisting >4 weeks. Additional details are in Table 2 and Extended Data Fig. 6.
Recruitment	Recruitment details for the acute cohort is detailed in O'Doherty et al., 2022. In short, hospital inpatients or from outpatient clinics >18 years old and PCR confirmed SARS-CoV-2 were recruited in St James's Hospital Dublin as part of the STTAR Bioresource, the aim of which is to create a biobank of samples during the COVID-19 pandemic in Ireland. For the long COVID cohort, recovered or long COVID participants >18 years old with a PCR confirmed SARS-CoV-2 infection were recruited for neuroimaging. Participants were excluded if they had a prior history of head trauma or other neurological disorder. Informed consent was obtained from each participant. All ethical approvals were in place prior to the initiation of studies on human subjects. All experiments conformed to the principles set out in the WMA Declaration of Helsinki and the Department of Health and Human Services Belmont Report. The St James' Hospital ethics committee approved these studies.
Ethics oversight	St James's Hospital ethics committee.

Note that full information on the approval of the study protocol must also be provided in the manuscript.

Field-specific reporting

Please select the one below that is the best fit for your research. If you are not sure, read the appropriate sections before making your selection.

☒ Life sciences ☐ Behavioural & social sciences ☐ Ecological, evolutionary & environmental sciences

For a reference copy of the document with all sections, see nature.com/documents/nr-reporting-summary-flat.pdf

Life sciences study design

All studies must disclose on these points even when the disclosure is negative.

Sample size	Sample size was determined based on sample availability. Samples were collected during the initial wave of SARS-CoV-2 infection in 2020.
Data exclusions	Cytokine data was excluded if falling below the count threshold for the MAGPIX xPONENT software with a minimum count of 50 required per analyte. Two participants that underwent imaging did not have blood samples collected.
Replication	Cytokine data was replicated with additional multiplex kits or by ELISA. q-RT-PCR data was performed on a minimum of 3 biological replicates with success. Neuroimaging data was replicated in consecutive participants. Imaging experiments were not replicated in additional cohorts due to the nature of our cohort being vaccine naive, making replication impossible.
Randomization	Experimental groups were determined by symptom status of patients. Covariates were included in statistical tests and included age, sex and co-morbidities.
Blinding	Investigators were blind to group allocation.

Reporting for specific materials, systems and methods

We require information from authors about some types of materials, experimental systems and methods used in many studies. Here, indicate whether each material, system or method listed is relevant to your study. If you are not sure if a list item applies to your research, read the appropriate section before selecting a response.

Materials & experimental systems

n/a	Involved in the study
☐	☒ Antibodies
☐	☒ Eukaryotic cell lines
☒	☐ Palaeontology and archaeology
☒	☐ Animals and other organisms
☒	☐ Clinical data
☒	☐ Dual use research of concern
☒	☐ Plants

Methods

n/a	Involved in the study
☒	☐ ChIP-seq
☒	☐ Flow cytometry
☐	☒ MRI-based neuroimaging

Antibodies

Antibodies used

Alexa Fluor™ 488 Phalloidin was used to stain F-actin filaments
Invitrogen ICAM-1 Monoclonal Antibody (1A29), #MA5407 Invitrogen™ for blocking studies
MitoTracker™ Orange was used to stain PBMCs for adhesion assays
Rabbit anti-GFbeta Abcam, #ab92486
Anti GFAP monoclonal antibody Merck #G3893

Validation

Validation data for antibodies and reagents are on manufacturers website below: https://www.thermofisher.com/order/catalog/product/A12379 https://www.fishersci.se/shop/products/anti-cd54-clone-1a29-ma5407/11844311 https://www.thermofisher.com/order/catalog/product/M7510https://www.abcam.com/products/primary-antibodies/tgf-beta-1-antibody-ab92486.htmlhttps://www.sigmaaldrich.com/IE/en/product/sigma/g3893

Eukaryotic cell lines

Policy information about cell lines and Sex and Gender in Research

Cell line source(s)

hCMEC/d3 was purchased from Merck Millipore

Authentication

hCMEC/d3 was confirmed as brain endothelial with CD31 staining.

Mycoplasma contamination

All cell lines tested negative for mycoplasma contamination.

Commonly misidentified lines
(See ICLAC register)

No commonly misidentified cell lines were used in the study.

Magnetic resonance imaging

Experimental design

Design type

Dynamic contrast-enhanced magnetic resonance imaging

Design specifications

n/a

Behavioral performance measures

n/a

Acquisition

Imaging type(s)

Structural and contrast imaging

Field strength

3T

Sequence & imaging parameters

Imaging was performed with a 3T Philips Achieva scanner. Sequences included a T1- weighted anatomical scan (3D gradient echo, TE/TR =3/6.7 ms, acquisition matrix 268x266, voxel size: 0.83x0.83x.9mm), T2-weighted imaging (TE/TR =80/3000 ms, voxel size: 0.45x0.45x.4mm), FLAIR (TE/TR =125/11000 ms, voxel size:0.45x0.45x4mm). For the calculation of pre-contrast longitudinal relaxation time (T10), the variable flip angle (VFA) method was used (3D T1w-FFE, TE/TR = 2.78/5.67 ms, acquisition matrix: 240x184, voxel size: 0.68x0.68x5 mm, flip angles: 10, 15, 20, 25 and 30°). Dynamic contrast enhanced (DCE) sequence was then acquired (Axial, 3D T1w-FFE, TE/TR = 2.78/5.6 ms, acquisition

	matrix: 240x184, voxel size: 0.68x0.68x5 mm, flip angle: 6°, Tt = 6.5 Sec, temporal repetitions: 61, total scan length: 22.6 minutes).
Area of acquisition	Whole brain imaging

Diffusion MRI ☐ Used ☒ Not used

Preprocessing

Preprocessing software	Volbrain was used for volume and thickness measurements https://www.volbrain.net/<br>For DCE-MRI analysis, SPM12 was used for image segmentation and alignment
Normalization	Affine registration to MNI space
Normalization template	MNI152
Noise and artifact removal	For DCE-MRI, differences in baseline physiological responses such as heart rate and blood pressure were controlled by normalising contrast signal to the signal of the superior sagittal sinus for each participant. Motion artifacts were manually excluded from analysis.
Volume censoring	n/a

Statistical modeling & inference

Model type and settings	Mass univariate
Effect(s) tested	GLM of COVID vs non-COVID with age, sex and total intracranial volume as covariates.

Specify type of analysis: ☐ Whole brain ☐ ROI-based ☒ Both

Anatomical location(s)	DCE-MRI ROI analysis was performed by manually drawing ROIs on DCE-MRI output maps.<br>Volume/thickness measurements were performed as described on Volbrain site https://www.volbrain.net/
Statistic type for inference<br>(See Eklund et al. 2016)	Voxel-wise
Correction	FDR

Models & analysis

n/a	Involved in the study
☒	☐ Functional and/or effective connectivity
☒	☐ Graph analysis
☒	☐ Multivariate modeling or predictive analysis
